# Advanced electrocatalysts for fuel cells: Evolution of active sites and synergistic properties of catalysts and carrier materials

**DOI:** 10.1002/EXP.20230052

**Published:** 2024-06-10

**Authors:** Zhijie Kong, Jingcheng Wu, Zhijuan Liu, Dafeng Yan, Zhi‐Peng Wu, Chuan‐Jian Zhong

**Affiliations:** ^1^ Henan Key Laboratory of Crystalline Molecular Functional Materials Green Catalysis Center College of Chemistry Zhengzhou University Zhengzhou China; ^2^ Department of Chemistry State University of New York at Binghamton Binghamton New York USA; ^3^ Hubei Collaborative Innovation Center for Advanced Organic Chemical Materials Ministry‐of‐Education Key Laboratory for the Synthesis and Application of Organic Functional Molecules College of Chemistry and Chemical Engineering Hubei University Wuhan China; ^4^ KAUST Catalysis Center Physical Sciences and Engineering Division King Abdullah University of Science and Technology Thuwal Saudi Arabia

**Keywords:** active site, electrocatalyst, low/high‐temperature fuel cell, oxygen reduction reaction, synergistic property

## Abstract

Proton exchange‐membrane fuel cell (PEMFC) is a clean and efficient type of energy storage device. However, the sluggish reaction rate of the cathode oxygen reduction reaction (ORR) has been a significant problem in its development. This review reports the recent progress of advanced electrocatalysts focusing on the interface/surface electronic structure and exploring the synergistic relationship of precious‐based and non‐precious metal‐based catalysts and support materials. The support materials contain non‐metal (C/N/Si, etc.) and metal‐based structures, which have demonstrated a crucial role in the synergistic enhancement of electrocatalytic properties, especially for high‐temperature fuel cell systems. To improve the strong interaction, some exciting synergistic strategies by doping and coating heterogeneous elements or connecting polymeric ligands containing carbon and nitrogen were also shown herein. Besides the typical role of the crystal surface, phase structure, lattice strain, etc., the evolution of structure‐performance relations was also highlighted in real‐time tests. The advanced in situ characterization techniques were also reviewed to emphasize the accurate structure‐performance relations. Finally, the challenge and prospect for developing the ORR electrocatalysts were concluded for commercial applications in low‐ and high‐temperature fuel cell systems.

## Graphical abstract

This review describes the evolution of active sites and synergistic properties of catalysts and support materials. In terms of precious metal catalysts, the effects of coordination and synergistic effects, phase structure, crystal surface engineering, and lattice strain were introduced. In non‐precious metal catalysts, carbon‐based and M‐N‐C electrocatalysts are introduced. Special emphasis is placed on the synergistic strategy of metal‐based catalysts and carrier materials.

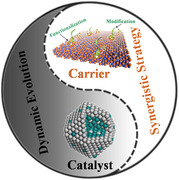

## 1 INTRODUCTION

PEMFCs are energy conversion and storage devices for mobile and stationary use with high energy density and zero emissions. However, the reaction kinetics of ORR at the cathode is much slower than the hydrogen oxidation reaction (HOR) at the anode. This is due to the need of high‐performance electrocatalysts for ORR to reduce the loading of Pt loading at cathodes, as Pt is expensive and rare.^[^ ^1^ ^]^ Developing advanced electrocatalysts for PEMFCs is a huge challenge. PEMFCs are distinguished into low‐temperature (LT‐PEMFC) and high‐temperature (HT‐PEMFC) based on their operating temperatures.^[^ ^2^, ^3^, ^4^, ^5^, ^6^, ^7^, ^8^, ^9^, ^10^ ^]^ The proton exchange membrane materials of the LT‐PEMFC and HT‐PEMFC system are respectively a polybenzimidazole membrane doped by phosphoric acid and a Nafion membrane composed of perfluorosulfonic acid (PFSA). Therefore, high‐performance acid‐resistant catalysts are critical for improving the activity and stability of acid LT‐PEMFC and HT‐PEMFC system. In the last decade, HT‐PEMFCs have gained more attention, but there are only a few reports on them.^[^ ^11^, ^12^, ^13^, ^14^, ^15^ ^]^ The design of the flow field for LT‐PEMFCs is complicated because the water vapor and liquid water are coexist, which makes water management a serious challenge. In addition, to prevent poisoning, the high‐purity hydrogen is needed in LT‐PEMFCs. Furthermore, the base metal catalysts in LT‐PEMFCs are prone to Fenton reactions due to the oxygen radical species caused by liquid H_2_O. However, HT‐PEMFCs are capable of resisting poisoning and do not require high‐purity hydrogen.^[^ ^14^, ^15^ ^]^ Improved heat and water management system, the capacity to avoid the Fenton reaction, and the high reaction kinetic make it possible to reduce Pt loading, even to the point of not using Pt at all.^[^ ^11^, ^16^ ^]^ This greatly increases the potential application of nonprecious‐based catalysts, particularly metal‐free carriers, in HT‐PEMFCs. For LT‐PEMFC, the development of Pt‐based alloy catalysts, especially the low‐Pt‐based catalysts, is the key issue for improving activity while reducing stability. However, for HT‐PEMFC with high temperature and strong phosphoric acid environment, it is particularly important to develop the anti‐poisoning of phosphoric acid, as well as corrosion‐resistant catalysts. Compared to previous studies,^[^ ^1^, ^6^, ^10^, ^15^, ^16^, ^17^, ^18^ ^]^ this review presents a universal solution to the problem of acidic proton membrane fuel cell catalysts of different systems, emphasizing the importance of the interaction between precious metal‐based catalysts and modified carbon‐based supports and pointing out that the development of both support and catalyst materials is the key to solving the problem of practical fuel cell catalysts.

The Department of Energy (DOE) has set performance targets for fuel cells. precious group metal‐free (PGM‐free) and PGM‐based catalysts must achieve mass activity of 0.044 A cm^−2^ and 0.44 A mg^−1^, respectively.^[^ ^19^ ^]^ High activity and stability of cathodic catalysts are needed to resolve the sluggish rate of ORR electrochemical reactions. Ideal catalyst properties are low cost, superior durability, and high inherent activity, determined by inherent material types.^[^ ^20^, ^21^ ^]^ PGMs are commonly used for commercial fuel cell electrocatalysts. ORR electrocatalysts have been widely researched in the last two decades using the rotating disk electrode (RDE) model testing system. Mass activities of 3‐13 A mg_Pt_^−1^ have been recorded.^[^ ^5^, ^6^, ^10^, ^22^, ^23^, ^24^ ^]^ In fuel cell technology, there is a gap in practical implementation. Two kinds of designing catalysts are PGM catalysts and nonprecious‐based catalysts. Engineering strategies such as surface strain, defect sites, and crystal facets have been reported to enhance their electrocatalytic activity for ORR.^[^ ^2^, ^3^, ^6^, ^10^ ^]^ Among them, carbon‐based loading materials are commonly used, but their interaction with PGM‐based catalysts is not well studied and there are still many challenges to overcome, such as metal structure change and carbon corrosion issues in the real reaction process.^[^ ^2^, ^4^, ^8^, ^15^ ^]^ Researchers found that using high surface area carbon carriers (HSC) can boost PtCo or PtNi activity in low‐temperature PEMFCs. Defect supports have strong interaction with metals, such as metal anchoring on carbon, positively affecting the ORR long‐term stability.^[^ ^25^, ^26^, ^27^, ^28^ ^]^

Understanding and constructing PGM‐based electrocatalysts and carriers is still a challenge, especially for their synergetic effects. Ordered Pt alloy catalysts can provide a promising solution strategy. Li et al. and Sun et al. reported good activity and stability of ordered Pt‐based catalysts on the membrane assembling electrode (MEA) under HT‐PEMFC and LT‐PEMFC, respectively. Moreover, the precious group metal‐based catalysts and carbon‐based carrier materials with better acid and heat resistance can solve challenges faced by high and low‐temperature PEMFCs, whose strong interaction is essential for making universal catalysts for both types of fuel cells.^[^ ^29^, ^30^ ^]^ Herein, we provide an overview of the synergy strategy between PGM‐based and loading carriers for the HT‐PEMFC and LT‐PEMFC, including their active sites, catalytic mechanisms, and design strategies. Some novel supporting materials and synergetic strategies were discussed to enhance the performance. In situ characterization technologies are demonstrated to understand the structure‐performance relationship during accelerated durability testing (ADT) more clearly. We also provide insights into future development directions by reviewing the remaining challenges.

## 2 ACTIVE SITES OF PGM‐BASED AND CARBON‐BASED CATALYSTS

Understanding active sites for ORR is vital for designing high‐performance catalysts. The ORR can follow either a four‐electron pathway [O_2_ + 4 (H^+^ + e^−^) → 2 H_2_O], producing water and benefiting fuel cells, or a two‐electron pathway, resulting in the production of hydrogen peroxide which is not favorable for fuel cells. During the process, optimal interaction between adsorbed substances and catalysts is crucial for enhancing ORR activity. Strong bonding energy between electrocatalyst and adsorbed oxygen (O_ad_) suppresses activity by facilitating electron transfer to O_ad_ or OH_ad_. To enhance ORR performance, design electrocatalysts with optimal interaction between O_ad_ and the active site.^[^ ^31^ ^]^ A model was developed to compute the free energy of the corresponding intermediates via functional density function calculation (DFT) proposed by Nørskov et al. The volcano‐shaped model explains why Pt is a preferred ORR catalyst, and why its performance could be enhanced by alloying Pt.^[^ ^31^, ^32^, ^33^, ^34^ ^]^ For precious metal Pt‐based catalysts, the Pt atomic site is the most critical catalytic active site for both HT‐PEMFC and LT‐PEMFC.^[^ ^4^, ^23^, ^24^, ^35^ ^]^ The DFT displays the correlation between O binding energy and activity. The volcano shape reveals that Pt is the best catalyst for oxygen reduction among most of the elements, in line with previous experiments. Moreover, metals with lower oxygen binding energies than Pt have a higher rate of oxygen reduction. Pt alloys with Ni, Co, Fe, etc. have smaller oxygen binding energies than pure Pt, according to DFT calculations. However, O_ad_ and OH_ad_ binding energies are roughly linearly correlated for elemental surfaces, and OH_ad_ binding energies are not reduced to the same extent as O_ad_ binding energies on these surfaces. The results show that compared with pure Pt, 3d transition metal has lower O_ad_ binding energy after alloying with Pt, which should give a higher reactivity.^[^ ^31^ ^]^

Carbon‐based metal‐free electrocatalysts' active sites for the ORR are determined by spin density and atomic charge.^[^ ^36^, ^37^ ^]^ Doping sp^2^ carbon with N atoms disrupts electrical neutrality, enhancing the density of the asymmetric spin distribution on the adjacent carbon atoms. This promotes O_2_ adsorption, which is beneficial to ORR.^[^ ^38^, ^39^, ^40^, ^41^, ^42^ ^]^ Other strategies were also constructed to activate inserted sp^2^ carbon into high‐performance active sites for ORR electrocatalysts, such as defect construction, and synergetic role with metal.^[^ ^43^, ^44^, ^45^, ^46^, ^47^, ^48^, ^49^, ^50^, ^51^ ^]^ Zhou investigated the impact of various types of N‐doping on the activity of defective carbon. It has been found that pyridine‐N contributes to ORR performance higher than graphite‐N.^[^ ^52^, ^53^, ^54^, ^55^ ^]^

All in all, metal‐free carbon coupled with multiple dopants has shown promising results in ORR.^[^ ^47^, ^48^, ^49^, ^56^, ^57^, ^58^, ^59^, ^60^ ^]^ Designing high synergetic strategies for PGM‐based and non‐metal carrier catalysts should be considered to reduce PGM content and enhance ORR performance.

## 3 PERFORMANCE CONTROL STRATEGY IN PGM (Pt/Pd‐BASED) ELECTROCATALYSTS

Pt‐based catalysts are commonly used in practical fuel cells. Designing high‐performance electrocatalysts requires two methods, including the adsorption site and electronic structure. The electronic structure can be engineered by controlling surface strain, defect site, and crystal facet.^[^ ^61^, ^62^, ^63^, ^64^ ^]^ Doping strategy is popular for modifying the adsorption site,^[^ ^65^, ^66^ ^]^ which will be discussed in the following sections.

Noble metal‐based nanomaterials are efficient fuel cell electrocatalysts in acidic electrolytes. The relationship between their structure and electrocatalytic performance has been well‐documented.^[^ ^67^, ^68^, ^69^, ^70^, ^71^, ^72^, ^73^ ^]^ Few studies have focused on the structural evolutions during the electrochemical test process. Aided by ex situ and especially in situ/in operando techniques, this section discusses the catalyst structure evolution and its impact on electrocatalytic performance, focusing on noble metal‐based nanoparticles and nanowires for ORR applications. Recent catalyst design and manipulation examples are briefly introduced for ORR. PGM‐based nanoparticles are promising ORR electrocatalysts. However, their stability in the practice operating condition of fuel cells is a crucial issue that hinders their mass commercialization. Understanding the morphology and composition‐structure evolution of the electrocatalysts is crucial.

### 3.1 Coordination and synergistic effects: Morphology and composition evolution

Transition metals are added into Pt atoms to form alloys, which could modify the electrocatalyst's surface catalytic site and electronic structure, known as the ligand effect.^[^ ^64^ ^]^ Influenced by both the adsorbate (O_ad_) and the d‐band center, the binding energy could be modified for PGM‐based alloy.^[^ ^74^ ^]^ To optimize the adsorption and desorption process in oxygen reduction reaction, the Fermi level and the d‐band center are always proposed to pursue an appropriate distance, which affects the binding energy of adsorbate, such as the coverage of OH_ad,_ the too‐strong or too‐weak binding energy of adsorbate on the active sites could limit the ORR rate. Thus, balancing oxygen adsorption and oxygenated species is crucial for enhancing ORR performance. Studies have shown that alloying Pt with metals such as Pd, Ni, and Au can significantly enhance the ORR process.^[^ ^75^, ^76^, ^77^, ^78^, ^79^ ^]^ Additionally, Pt‐based nanowires proposed by Kong et al.,^[^ ^23^ ^]^ and PtPdCu ternary composition alloy catalysts revealed by Wu et al., have been discovered with distinguished durability despite the occurrence of dealloying.^[^ ^35^ ^]^

The influence of temperature change on the structure and properties of the catalyst was studied by Wang et al.^[^ ^80^ ^]^ The process of hydrogen intercalation and exsolution in the Pd lattice interstitial sites was studied by using in situ temperature‐variable XRD, TPD, and TPSR experiments. The results showed that hydrogen atoms spontaneously entered the Pd lattice interstitial sites at room temperature, and as the temperature increased, hydrogen on the subsurface gradually released. Between 25 and 100°C, the hydrogen atoms remained retained in Pd, while at 160°C, the hydrogen atoms embedded in the subsurface lattice interstitial sites were released. The TPD and TPSR experiments demonstrated that the hydrogen atoms released from Pd/C followed two procedures. The hydrogen atoms in the subsurface layer began to escape at about 106°C, while those in the inner layer started escaping at about 300°C. Compared with the Ar environmental control experiment, it verifies that the shift of the XRD peak position was caused only by the permeated hydrogen atoms into the interstitial sites of Pd/C at 25–300°C.^[^ ^80^ ^]^

**FIGURE 1:**
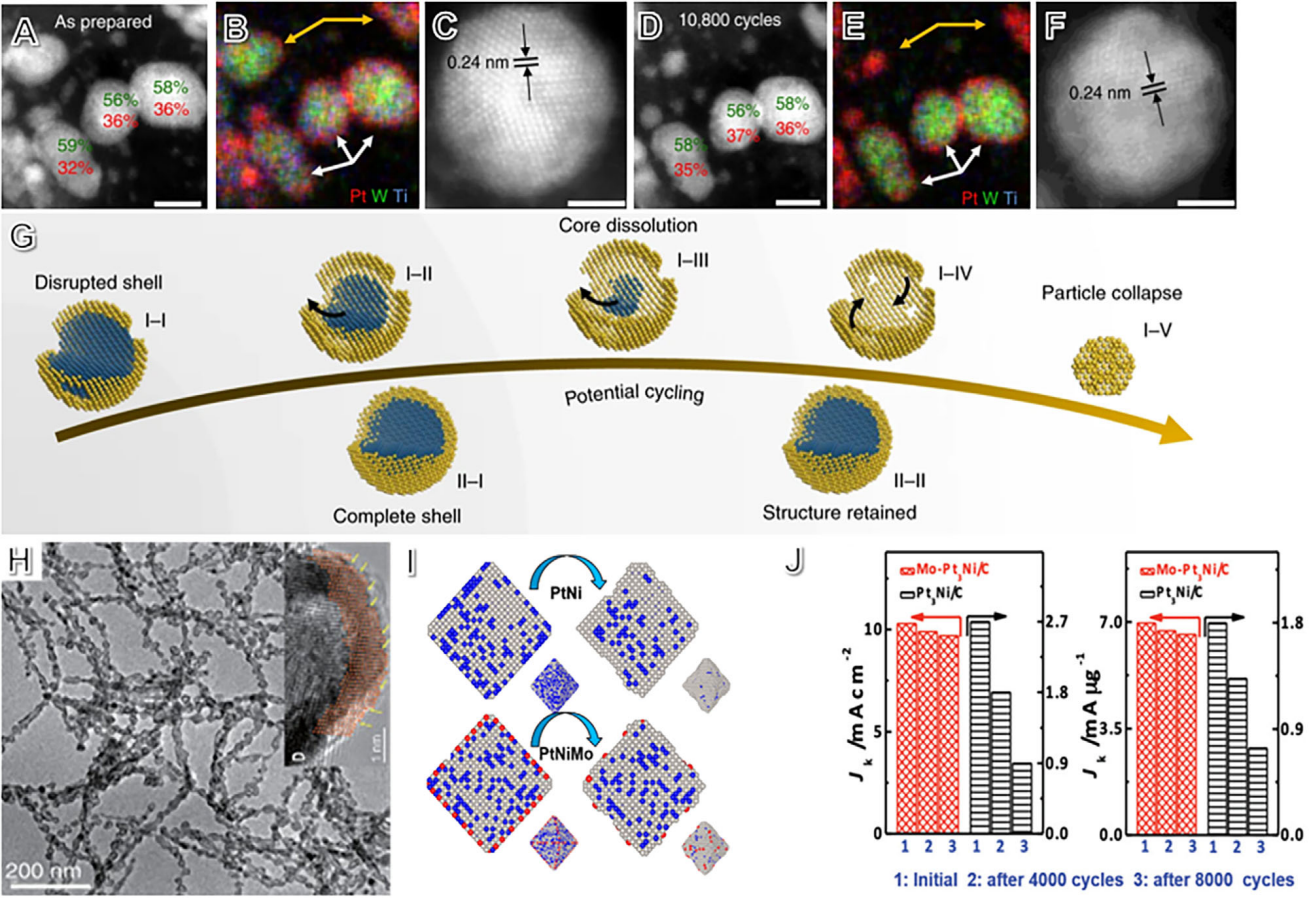
Coordination and synergistic effects of Pt‐based electrocatalysts on fuel cells. STEM images of TMC@Pt NPs were captured (A–C) before and (D–F) after durability tests. (G) The evolution process of partially and fully core@shell NPs with Pt‐shell during long‐term cycling in the schematic illustration. Reproduced with permission.^[^ ^81^ ^]^ Copyright 2020, Nature Publishing Group. (H) TEM and HRTEM images of PtNi NWs with bunched alloy nanocages and defects. Reproduced with permission.^[^ ^86^ ^]^ Copyright 2019, AAAS. (I) The 3D RMC models of PtNi and Mo‐doped PtNi NPs during the accelerated durability test. (J) The performance of octahedral PtNi/C and Mo‐PtNi/C regarding MAs and SAs at 0.9 V (vs RHE). Reproduced with permission.^[^ ^87^ ^]^ Copyright 2015, AAAS.

The recent study conducted by Göhl's group investigated the changes in the structure and composition of Pt‐based ORR electrocatalysts during the in situ test at room temperature. The study used identical location‐STEM coupled with the elemental mapping of energy dispersive X‐ray (EDX) and in situ ICP mass spectrometry (ICP‐MS) techniques. Specifically, the study focused on a phase‐segregated core–shell NP with a carbon‐based transition metal core coated with a Pt atom layer (TMC@Pt).^[^ ^81^ ^]^ To better understand the durability of the TMC@Pt structure, different Pt atom‐layer coatings were created, from partial shell coverage to complete shell coating. Figure 1A–G shows the identical location‐STEM/EDX images demonstrating the TMC@Pt catalyst with a complete core@shell maintains its core–shell characterization and similar particle size in long‐term testing. However, the TMC@Pt NPs dissolve gradually in the durability test, resulting in a Pt‐rich and hollow particle collapse process and a much smaller particle size. In situ ICP‐MS experiments revealed a slower core element dissolution rate from the fully‐covered TMC@Pt NPs than the partially‐coated counterpart, further substantiating their structural stability. This study raises questions about the structural stability of Pt‐based core–shell structural NPs with Pt or Pt‐rich shell and demonstrates distinguished electrochemical durability due to their robust Pt‐shell. By the way, such core‐shell structures can be fabricated using seed‐mediated growth,^[^ ^82^ ^]^ underpotential deposition followed by chemical etching, galvanic displacement,^[^ ^83^ ^]^ thermochemical and absorbate‐induced annealing, etc., and demonstrate distinguished electrochemical durability due to their robust Pt‐shell structure.

Defect engineering is crucial for adsorption site modification. Bandarenka et al. proposed a generalized coordination number (CN) to describe the relation of targeted and neighboring atoms for ORR performance.^[^ ^66^ ^]^ The concave defective site of dendritic surface structure improves ORR activity. Concave defect sites (CN > 7.5) demonstrate a weaker adsorption bond for *OH than that on Pt (111), demonstrating superior performance.^[^ ^84^, ^85^ ^]^ Maillard et al. introduced “Surface Distortion” (SD) to evaluate the relations between ORR performance and catalyst structure. PGM‐based defective catalysts are gaining much research attention to commercialize catalysts for both half‐cell and whole‐cell systems.^[^ ^4^ ^]^ For example, the Xia group created a Pt‐rich Pt–Ni nanowire catalyst with connected cage morphology called PtNi‐BNS,^[^ ^86^ ^]^ with a unique defect structure on the surface (Figure 1H). After testing them in hydrogen‐air fuel cells, the PtNi‐BNSs had a higher power density and showed negligible decay during a 180‐h durability test. These nanowires are a promising option for the application in hydrogen‐air fuel cells.

The above alloying PGM metal is a bulk ligand engineering strategy. Surface ligand element doping on PGM‐based alloy nanomaterials creates defective sites, which is viewed as a location doping strategy. Huang's team used this strategy to synthesize surface‐doped Mo on Pt_3_Ni (Mo‐Pt_3_Ni). The study shows that Mo doping protects base metal leaching from Mo‐PtNi/C, leading to the same content of subsurface Ni under the electrochemical process, thus obtaining high durability. Calculations by the corresponding 3D reverse Monte Carlo model (RMC) (Figure 1I) demonstrated that the activity was improved due to the decreased energy of neighboring atoms with doped Mo atoms, thus protecting the other base metal leaching out on the vertex or edge site. Compared with the initial activity (10.3 mA cm^−2^_Pt_), the Mo‐PtNi nanoparticles still maintained a high electrocatalytic activity of 9.7 mA cm^−2^_Pt_ after 10,000 cycles of electrochemical durability test (Figure 1J), which suggests that appropriate Mo atom doping can improve the stability of octahedral PtNi catalysts.^[^ ^87^ ^]^ By using this near‐surface doping strategy, the activity or stability of many Pt‐base alloy electrocatalysts has been significantly improved.^[^ ^24^, ^88^, ^89^, ^90^, ^91^ ^]^ Doping has the capacity to alter the surface composition and modify the adsorption sites, thereby improving the ORR performance. The catalytic performance is significantly affected by different components and morphologies via alloying and doping strategies.

### 3.2 Phase structure engineering

**FIGURE 2:**
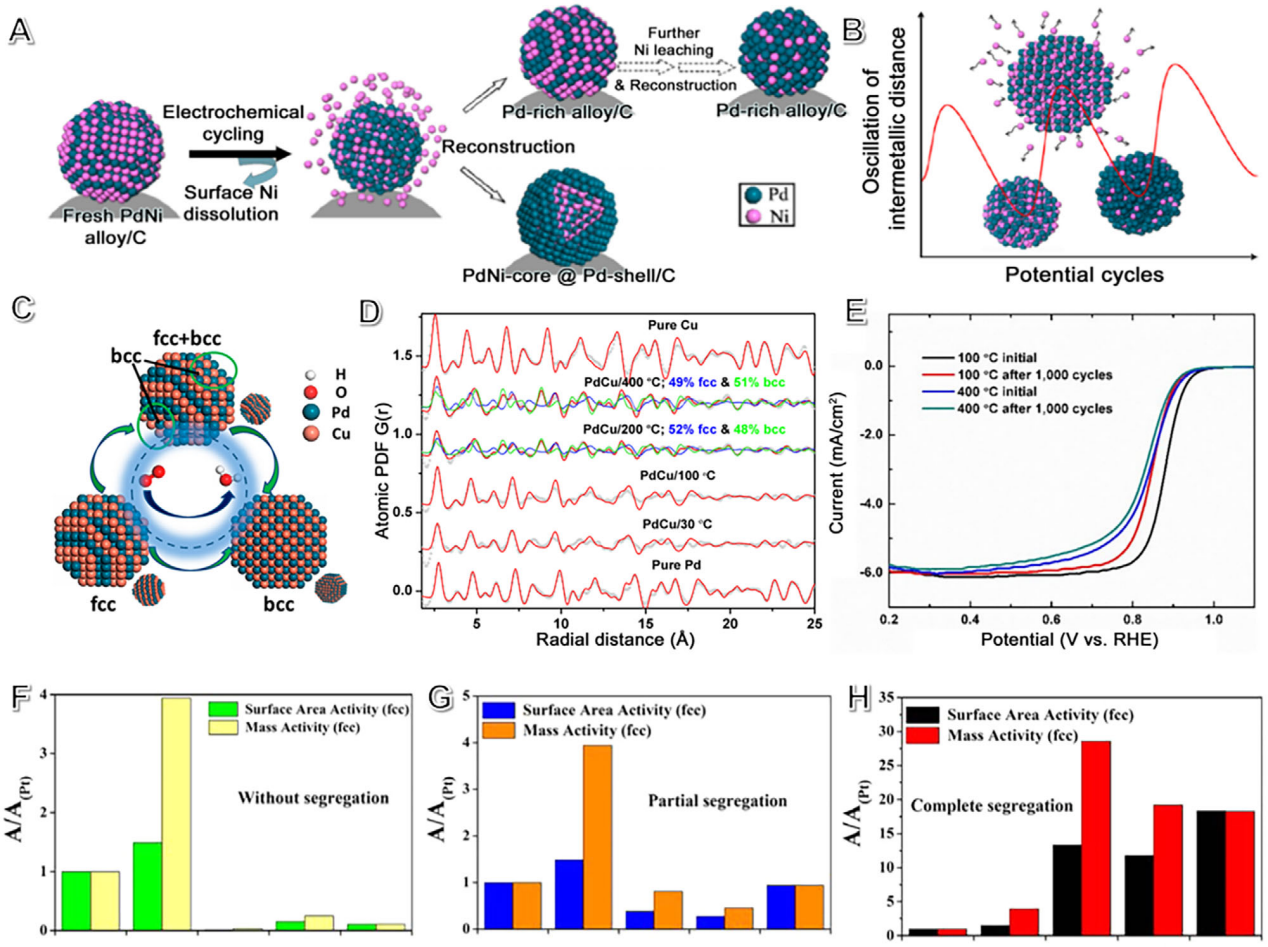
The role of phase structure engineering of Pt‐based electrocatalysts on fuel cells. (A) The schematic diagram of the structural changes in the PdNi/C NP electrocatalyst during the ADT test. (B) Intermetallic distance oscillatory phenomenon versus the potential cycles. Reproduced with permission.^[^ ^94^ ^]^ Copyright 2015, American Chemical Society. (C) Illustration of phase structure changes in PdCu nanoparticles. (D) Atomic PDFs from crystal structure models and experiments of various catalysts. (E) ORR curves of Pd_50_Cu_50_/C‐400°C and Pd_50_Cu_50_/C‐100°C catalysts in the ADT process. Reproduced with permission.^[^ ^95^ ^]^ Copyright 2018, American Chemical Society. The relationship between performance and the degree of phase segregation of Pd_(1‐_ *_x_*_)_Au_(_ *_x_*_‐_ *_y_*_)_Pt*_z_* @AuyPt_(1‐_ *_z_*_)_ NW, related to the fcc sites, (F) without segregation, (G) partial segregation, (H) completed segregation. The *X*‐axis from left to right is Pt, Pt@Pd, Pd_0.88_Pt_0.19_ @Au_0.12_Pt_0.81_, Pd_0.77_Pt_0.38_@Au_0.23_Pt_0.62_, Pd_0.69_Pt_0.52_@Au_0.31_Pt_0.48_. Reproduced with permission.^[^ ^99^ ^]^ Copyright 2015, American Chemical Society.

Due to the inevitable evolution of components and structures in the electrochemical environment, the study of phase structure engineering and the phase structure evolution process has received more and more attention. Compared with the octahedron PtNi/C, sponge PtNi/C lost significant Ni during potential cycling, resulting in an increased lattice constant. In contrast, octahedron PtNi/C showed high structural stability. Despite the expectation that octahedron PtNi/C would have better ORR stability, the mass and specific activity trends showed contradictory results with potential cycling.^[^ ^92^ ^]^ Synchrotron HE‐XRD experiments were used to investigate the structure evolution process of PGM‐based nanoparticles during electrochemical potential cycling.^[^ ^93^, ^94^, ^95^, ^96^, ^97^ ^]^ The study focused on the PdNi/C ORR electrocatalyst and found that significant Ni dissolution occurred during the initial potential cycles, resulting in quick activity loss. The activity of the electrocatalyst mildly decreased due to the increasing NP size, leading to fewer exposed surface active sites. RMC models supported the reliability of both in‐situ and ex‐situ data.^[^ ^93^, ^94^ ^]^ The agglomeration and coarsening of NPs occurred due to dissolution/re‐deposition cycles, leading to considerable activity decay. To understand structure evolution during potential cycling, the atomic reconstruction dynamics were examined. PdNi/C NP electrocatalyst's intermetallic distance oscillates during cycling due to repeated atom dissolution and re‐deposition. Figure 2A,B shows the dynamic structure evolution for the lattice constant and intermetallic distance oscillation process of the PdNi/C nanocatalyst. After base metal leaching, two possible scenarios arise the PdNi alloy formation on the surface or a PdNi@Pd‐shell structure. It is unclear which structure shows at equilibrium. A recent study shows the surface structure evolved to an alloy state as an active site for ORR. We hypothesize the existence of “self‐healable” PGM‐based alloy electrocatalysts for stable operation in PEMFC.

PdCu/C NPs is a well‐studied PGM‐based alloy electrocatalyst. In a recent study, it was found that Pd_50_Cu_50_ had the highest mass activity for the ORR.^[^ ^98^ ^]^ Wu et al. examined the effects of treatment temperature under H_2_ atmosphere for the NP phase structure and corresponding electrocatalytic performance.^[^ ^95^ ^]^ The achieved phase structure manipulation between face‐centered cubic (fcc) and body‐centered cubic (bcc) of Pd_50_Cu_50_/C NP electrocatalysts (Figure 2C) via various annealing temperatures and was determined by HE‐XRD/PDF experiments. The Pd_50_Cu_50_/C NP treated at 100°C had a pure fcc phase, while the sample annealed at 400°C had a fcc‐ and bcc‐phase mixed structure (Figure 2D). The electrocatalytic performance of ORR was evaluated in the electrochemical cell. Pd_50_Cu_50_/C‐100°C initially exhibits high ORR mass activity but has poor durability, while Pd_50_Cu_50_/C‐400°C demonstrates lower initial mass activity but offers better stability (Figure 2E).

The Wong and Adzic group reported ultrathin core‐shell Pt‐Pd_9_Au segregation nanowires. The active structure for the ternary nanowires is a PtAu–Pd configuration. During the ORR, the adsorption of oxygen species could cause surface elemental reconstruction of Pt‐based alloys, which favored surface elemental segregation for nano‐catalysts. The level and distribution of Au within AuPd@Pt nanowires significantly impact their ORR activity. Au‐segregated nanowires Pd_(1‐_ *_x_*_)_Pt*_z_* @Au*_x_* Pt_(1‐_ *_z_*_)_ with complete Au segregation in their shell have higher mass and specific activities than pure Pt nanowires. On the other hand, partial Au‐segregated Pd_(1‐_ *_x_*_)_Au_(_ *_x_*_‐_ *_y_*_)_Pt*_z_* @Au*_y_* Pt_(1‐_ *_z_*_)_ nanowires and Pd_(1‐_ *_x_*_)_Au*_x_* @Pt nanowires with Au localized in the core have much lower ORR activities. Therefore, the distribution of Au within the nanowires plays a critical role in determining their ORR activity (Figure 2F–H).^[^ ^99^ ^]^ Optimal surface segregation variation is also a preferred way to engineer the phase structure.

### 3.3 Crystal facet engineering

**FIGURE 3:**
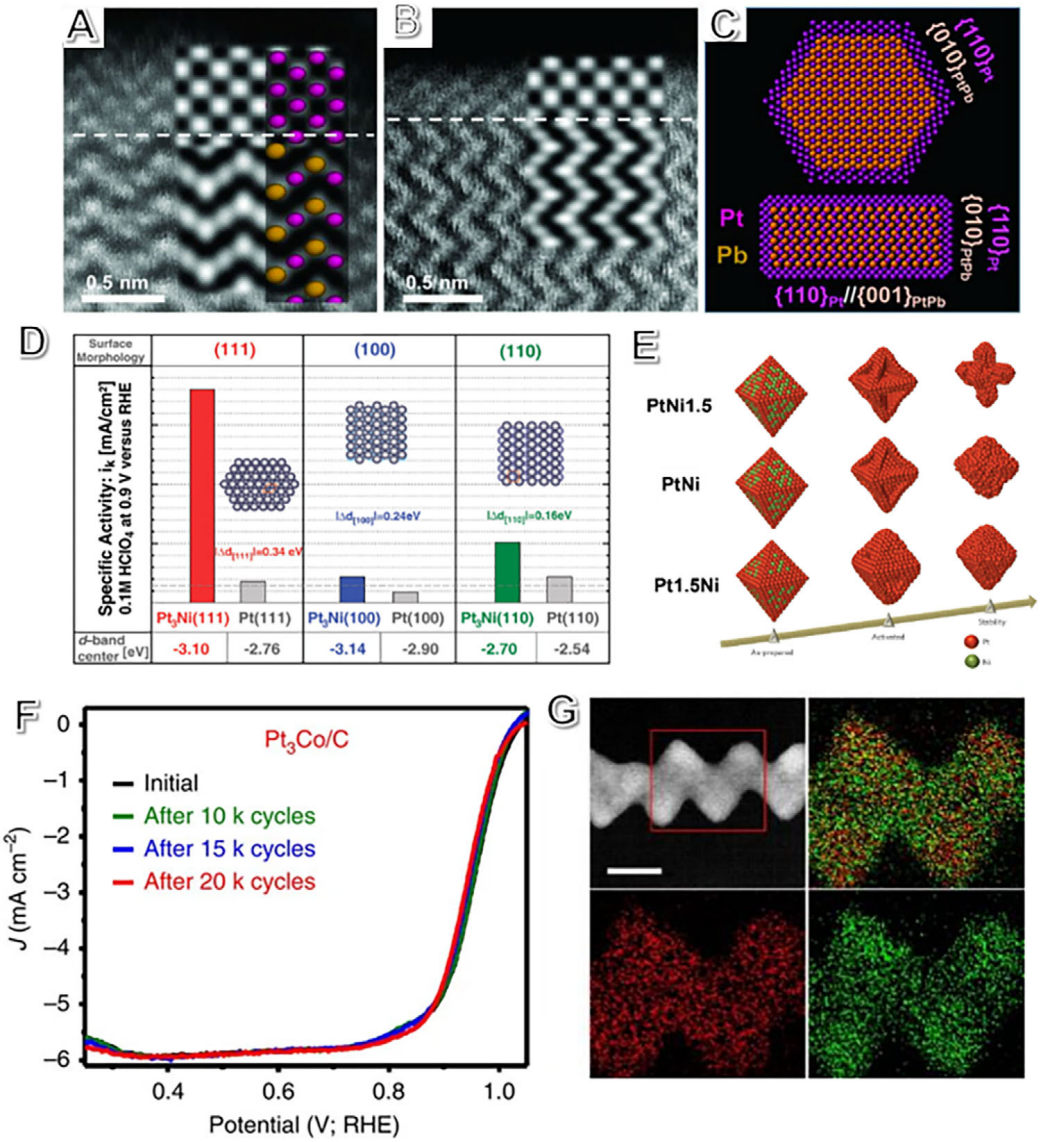
The role of crystal facet engineering of Pt‐based electrocatalysts on fuel cells. (A,B) The high resolution of HAADF images of single hexagonal PtPb nanoplates superposed with atomic models and simulated images on the experimental images. (C) The nanoplate illustrations demonstrate the top interface [(110)Pt//(100)PtPb] and side interface [(110)Pt//(001)PtPb]. (A‐C) Reproduced with permission.^[^ ^100^ ^]^ Copyright 2016, AAAS. (D) The d‐band center position obtained from the UPS spectrum based on the surface facet of Pt(hkl) and Pt_3_Ni(hkl). Adapted with permission.^[^ ^61^ ^]^ Copyright 2007, AAAS. (E) The changes of composition and morphology for Pt*_x_* Ni_1‐_ *_x_* NPs. Reproduced with permission.^[^ ^104^ ^]^ Copyright 2013, Springer Nature. (F) Polarization curves of the Pt_3_Co hierarchical NWs/C catalyst for ORR in 10,000, 15,000, and 20,000 accelerated durability experiments in different potentials versus RHE. (G) Images of HAADF‐STEM and the corresponding EDS of the Pt_3_Co hierarchical NWs. Reproduced with permission.^[^ ^105^ ^]^ Copyright 2016, Springer Nature.

The types of crystal faces and the spacing between crystal faces play an important role in regulating ORR adsorption and desorption. The Pt (110) facet in a nanoplate morphology is found by Bu et al. with a high ORR mass activity of 4.3 A mg_Pt_^−1^ and a high specific activity of 7.8 mA cm^−2^ at 0.9 V. PtPb/Pt nanoplates show a negligible drop after durability test.^[^ ^100^ ^]^ High‐resolution images of HAADF‐STEM coupled with the atomic models and schematic images revealed different PtPb and Pt phases stacking them in different ways, verifying the PtPb(hexagonal)/Pt(cubic) core/shell structure (Figure 3A–C). Compared with the {001}PtPb//{110}Pt and {010}PtPb//{110}Pt interfacial planes, which revealed the improved ORR activity due to the higher activity for Pt {110} facets than the Pt {111} crystal facet.^[^ ^100^, ^101^, ^102^ ^]^

The electronic structure of electrocatalysts can be adjusted by rearranging the atomic structure.^[^ ^61^, ^103^ ^]^ The Pt_3_Ni (111) single‐crystal catalyst synthesized by Marković et al. has state‐of‐the‐art specific activity and is better than Pt_3_Ni (110) and Pt_3_Ni(100) (Figure 3D).^[^ ^61^ ^]^ Pt*_x_* Ni_1‐_ *_x_* NPs with Ni‐rich PtNi (111) facet play a crucial role in enhancing the ORR activity (Figure 3E). However, the activity drops noticeably along with the structure evolution and component leaching, suggesting the stable role of the alloy (111) facet.^[^ ^104^ ^]^

Huang's group found that PtCo nanowires have high‐index facets and ordered intermetallic structures that increase their mass activity and specific activity for ORR, making them superior to commercial Pt/C catalysts (33.7 times). The Pt_3_Co nanowires maintain their composition, morphology, and structure even after undergoing 20,000 potential cycling cycles. The corresponding mass activity decreases only around 9% (Figure 3F,G).^[^ ^105^ ^]^ Similarly, Pt_3_Fe nanowires also have high‐index (311) facets and retain their composition even after 50,000 potential cycles.^[^ ^106^ ^]^

### 3.4 Lattice strain engineering

Designing catalysts with appropriate compressive strain is significant in studying the role of strain in the ORR process. DFT calculations explain complex phenomena and play an important role in systematically studying the quantitative relationship between catalyst lattice strain and ORR activity. The strain‐activity “volcano” curve is presented by the Strasser group,^[^ ^107^ ^]^ suggesting that compressive strain could significantly enhance ORR performance.

**FIGURE 4:**
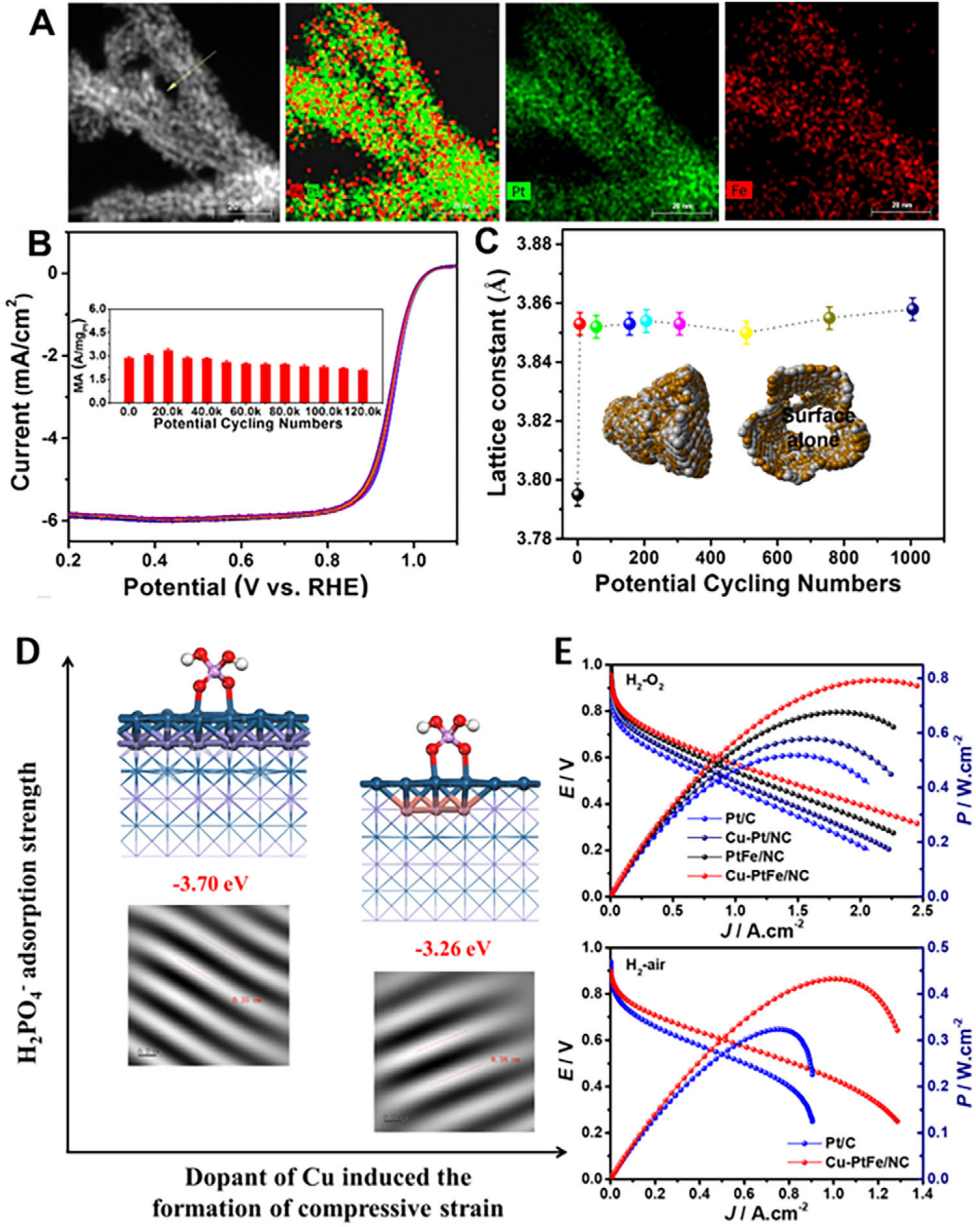
Effect of lattice strain on the performance of fuel cells. (A) EDX mapping and HAADF images for twisty PtFe NWs, (B) the ORR polarization curves and MAs in the ADT process (insets). (C) the relationship between the lattice constant and potential cycling number in PEMFCs over in situ synchrotron XRD, combined with the RMC model (insets). Reproduced with permission.^[^ ^23^ ^]^ Copyright 2020, American Chemical Society. (D) The relationship between H_3_PO_4_ adsorption and the compressive strain induced by Cu dopant. (E) The power density of Cu‐PtFe/NC in H_2_‐O_2_ and H_2_‐air.^[^ ^30^ ^]^ Copyright 2021, John Wiley and Sons.

Lattice expansion causes a decrease in bandwidth while shrinking leads to the downshifting of the anti‐bond state for transition metal and O_ad_.^[^ ^61^, ^107^ ^]^ Lattice strain engineering is thus applied for ORR. Kong et al. revealed the low‐Pt‐content PtFe_3_ twisty NWs and found around 70% activity after 120,000 potential cycles. They verified the lattice strains and structures using in‐situ and ex situ HE‐XRD, PDFs, and reverse Monte Carlo models. They also found that lower lattice strain coupled with the single alloy phase in Pt_24_Fe_76_ NWs than in Pt_42_Fe_58_ NWs contributed to enhanced ORR activity and durability (Figure 4A–C).^[^ ^23^ ^]^ The work emphasizes the key role of initial compressive strain in low‐Pt‐content nanoalloy systems.

Li et al. found that adding a small amount of Cu to PtFe alloys can enhance their ability to tolerate phosphate in the HT‐PEMFC system.^[^ ^30^ ^]^ This is achieved through a strain strategy that weakens the adsorption energy of phosphoric acid on Pt surfaces (Figure 4D). These electrocatalysts have achieved a distinguished power density of 432.6 and 793.5 mW cm^−2^ in H_2_‐air and H_2_‐O_2_ respectively, which is ~1.34 and 1.53 times higher than commercial Pt/C (Figure 4E).^[^ ^30^ ^]^ They also exhibit distinguished stability under the H_2_‐O_2_ atmosphere in the practice HT‐PEMFC, with negligible performance drop in 100 h, which verified the ordered catalyst's high‐temperature and phosphoric acid toxicity resistance. Sun et al. reported that the order structure significantly enhances the catalytic activity of Pt for ORR in 0.1 M HClO_4_ at both room temperature and 60°C and in MEA at 80°C. Due to the desired compressive strain of thin Pt shell caused by the Fe, The L10‐FePt/Pt ordered catalyst demonstrates a mass activity of 0.7 A mg_Pt_^−1^ in the half‐cell ORR test and shows no perceptible loss in mass activity after 30,000 potential cycles between 0.6 and 0.95 V at 80°C in the MEA. Thus, developing orderly Pt‐based alloy catalysts with compressive strain is an effective solution to solve the problems of these two kinds of PEMFCs simultaneously.^[^ ^29^ ^]^

Nanoparticles often have core–shell structures or twin defects that strain the nanocatalyst's surface.^[^ ^108^, ^109^ ^]^ To improve ORR performance, icosahedral Pt‐M (M = Au, Ni, Pd) nanoparticles with high‐density twin defects and high lattice strain were developed.^[^ ^110^ ^]^ Xia's team created a Pd@Pt concave decahedron, producing a suitable compressive strain thus leading to a high ORR activity.^[^ ^111^ ^]^ Even if the alloy structure is completely de‐alloyed, strain can still remain. Goddard group reported that during dealloying, Ni leaching from PtNi nanoparticles produced a high‐Pt‐content (Pt_85_Ni_15_) nanoporous structure with a compressive surface,^[^ ^112^, ^113^ ^]^ which explained the distinguished activity.

## 4 PERFORMANCE CONTROL STRATEGY IN PGM‐FREE ELECTROCATALYSTS

### 4.1 Metal‐containing (M‐N‐C) electrocatalysts

**FIGURE 5:**
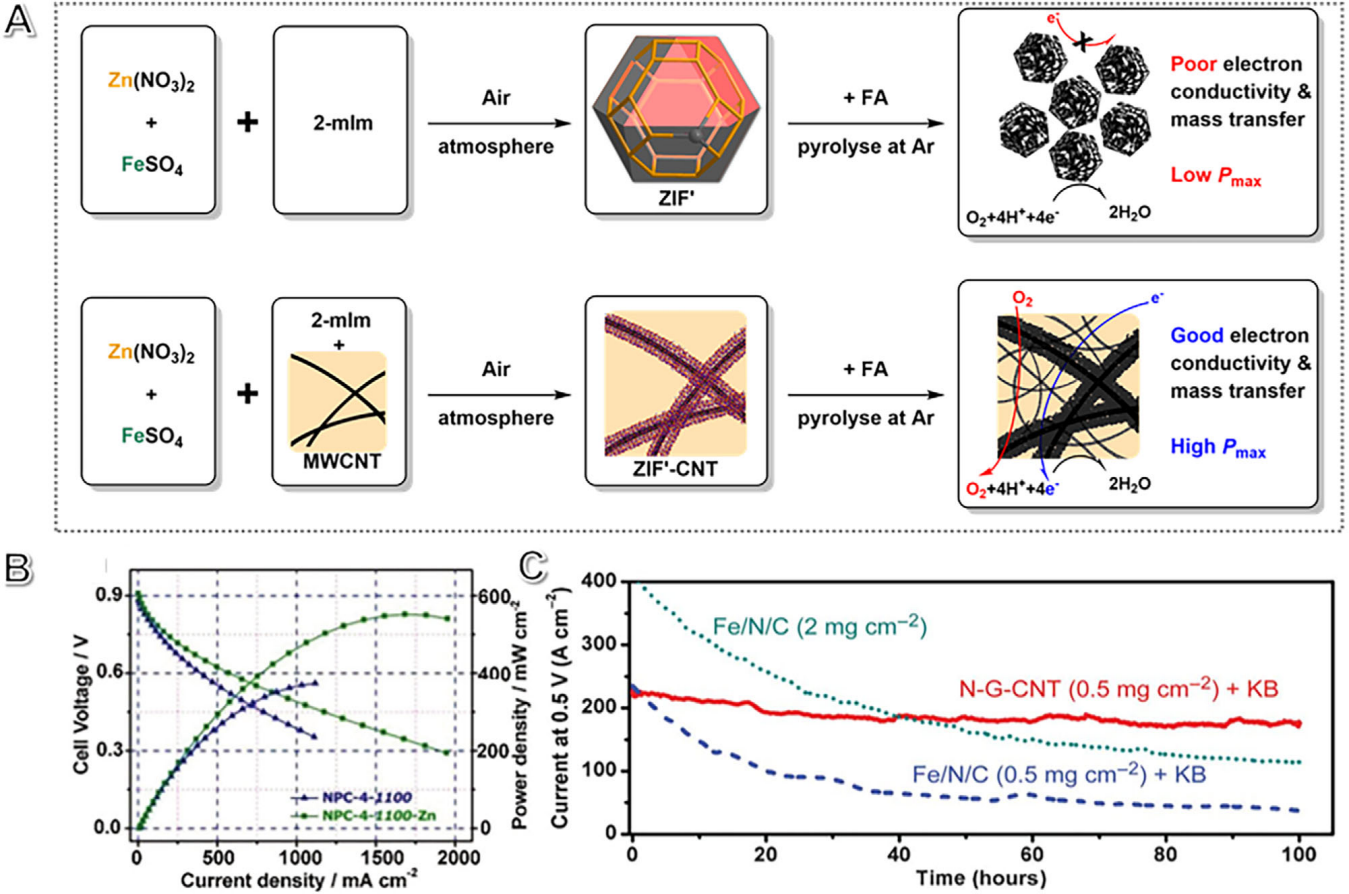
The role of metal‐containing (M‐N‐C) electrocatalysts on fuel cells. (A) The multiwalled carbon nanotube (MWCNT) was added to connect the NPs and enable electron conduction during the ZIF preparation. Reproduced with permission.^[^ ^117^ ^]^ Copyright 2016, John Wiley and Sons. (B) The performance of NPC‐4‐1100‐Zn and NPC‐4‐1100 electrocatalysts in PEMFC cells. Reproduced with permission.^[^ ^124^ ^]^ Copyright 2018, Elsevier B.V. (C) The accelerated test of the Fe/N/C catalyst and metal‐free N‐G‐CNT+KB over 0.5 V in PEMFCs. Reproduced with permission.^[^ ^129^ ^]^ Copyright 2015, AAAS.

Transition metal compounds containing C/N (named M‐C‐N) catalyst are promising catalysts, studied widely as a support or direct catalytic material. At moderate current densities in a fuel cell, voltage drops linearly due to ohmic loss caused by electronic and ionic resistances. M‐N‐C catalyst layers required for acceptable fuel cell performance are typically ten times thicker than Pt/C.^[^ ^114^ ^]^ M‐N‐C catalysts with a high loading of 3‐4 mg cm^−2^ are needed. The thicker M‐N‐C catalyst layer results in a larger ohmic loss due to the increased electronic resistance. The conductivity of M‐N‐C catalyst synthesis through high‐temperature pyrolysis (900‐1100°C) demonstrates distinguished differences.^[^ ^115^ ^]^ When the temperatures increase above 1100°C, ORR activity drops, making it an unrealistic strategy to increase the pyrolysis temperature.^[^ ^116^ ^]^ Incorporating conductive carbons, like carbon nanotubes, into M‐N‐C catalysts enhances their electronic conductivity.^[^ ^117^ ^]^ For instance, Zhang et al. proposed carbon nanotubes (CNTs) to crosslink catalyst nanoparticles, which exhibited a higher electronic conductivity than the one without CNTs, resulting in a decrease in the ohmic polarization loss (Figure 5A). The CNTs benefit from the increased electron conductivity and mass transfer. However, these additives can dilute the active sites of M‐N*_x_*, so non‐additive strategies have been developed. Shui et al. discovered a technique for creating an Fe‐N‐C catalyst with a network carbon nanofibrous structure that increased cell performance dramatically.^[^ ^118^ ^]^

Fuel cell performance depends on intrinsic activity, electronic resistance, and mass transfer. At high current densities, mass transfer dominates, causing concentration polarization loss and reducing performance. To minimize this loss, the M‐N‐C catalyst could be engineered to improve mass transport by creating a hierarchical porous structure.^[^ ^119^ ^]^ The template method can be used to increase mesopores, while porous carbon supports can enhance electronic conductivity. Using suitable N sources can also produce rich mesopores. Porosity is an essential characterization in M‐N‐C catalysts, whose active site is mainly located in the micropore. However, liquid water can easily block micropores, leading to flooding and hindering oxygen transport to the active site in pores.^[^ ^120^ ^]^ To enhance mass transport, building more macro/mesopores in M‐N‐C catalysts is an essential approach. Utilizing the template method is undoubtedly the most effective strategy to expertly modify M‐N‐C catalysts with pore structure. For example, Si and SiO_2_ were utilized to enhance the external surface area and mesopores of the Fe‐N‐C catalyst, thereby reducing the corresponding concentration in PEMFCs.^[^ ^121^, ^122^ ^]^ Porous carbon supports could enhance the number of mesopores or macropores, thus improving the electronic conductivity for M‐N‐C catalysts. Additionally, some N sources were used to obtain porous structures besides the controllable synthesis methods mentioned above. Using 2‐aminobenzimidazole as the N source, the Fe‐N‐C catalyst had more mesopores than that synthesized with m‐phenylenediamine, despite a similar BET surface area.^[^ ^123^ ^]^

Xing et al. obtained a record power density of 579 mW cm^−2^ using a N/P/Zn co‐doped carbon nanosheet (NPC‐4‐1100‐Zn) as metal‐free catalysts in a PEMFC (Figure 5B).^[^ ^124^ ^]^ The catalyst showed an onset potential of 0.91 V in 0.1 M HClO_4_, with a half‐wave potential of 0.79 V, which could be compared with M‐N‐C electrocatalysts. The distinguished performance is due to the carbon active site induced by N/P/Zn co‐doping, and the 3D microporous structure in a PEMFC. Most M‐N‐C catalysts under fuel cell systems have inferior durability due to the peroxide/free radical attack, demetallization, and irreversible inactivation of active sites protonation/anion.^[^ ^125^, ^126^, ^127^ ^]^ In comparison, carbon‐based, metal‐free catalysts have higher corrosion resistance. N‐doped carbon nanomaterials have large surface active sites, contributing to high performance and stability in acidic PEMFCs.^[^ ^127^, ^128^ ^]^ Dai and colleagues utilized Ketjen black carbon (KB) as a building block for creating N‐doped carbon materials, resulting in an electrode with higher durability and power performance than Fe/N/C catalyst (Figure 5C).^[^ ^129^ ^]^ N‐doped graphene with a rational 3D electrode configuration and an extensive range of active sites facilitated efficient gas and water transmission, enhancing CNT bone‐structure conductivity.

### 4.2 Metal‐free (carbon‐based) electrocatalysts

Pt‐based catalysts are widely carried by carbon‐based materials in PEMFCs.^[^ ^8^ ^]^ Besides the doping strategy of noble metal catalysts, doping engineering of non‐precious metal carriers is also conducive to improving apparent and intrinsic active sites in terms of constructing active sites. Defect catalysts can be developed through etching, doping, or dealloying.^[^ ^130^, ^131^ ^]^ Doping and etching are the most frequently used methods, with plasma etching being a common approach to obtain various defects.^[^ ^132^ ^]^ Heteroatomic doping, partially replacing local carbon atoms, can significantly increase activity. N doping is often accompanied by the absence of C atoms and the formation of holes or mesoporous structures. According to calculations based on quantum mechanics using B3LYP hybrid density functional theory, carbon atoms next to nitrogen dopants have a high positive charge density, which balances out the strong electronic affinity of the nitrogen atom. This mechanism is thought to facilitate the reduction of O_2_ on the carbon‐based metal‐free electrodes. These reduced carbon atoms are then re‐oxidized to their preferred oxidized state when O_2_ is absorbed. The parallel diatomic adsorption can weaken the O‐O bonding, making ORR at the N‐doped carbon‐based electrodes easier. Nitrogen heteroatom doping in carbon carriers, such as in the N‐doped carbon‐based electrodes, can efficiently create metal‐free active sites for electrochemical reduction.^[^ ^36^, ^133^ ^]^ The presence of both electron‐deficient and electron‐rich heteroatoms can enhance the performance by promoting the adsorption of oxygen.^[^ ^130^, ^131^ ^]^ Studies have shown that carbon‐based nanomaterials are promising alternatives to PGM catalysts due to their excellent electrical conductivity, low cost, and tolerance to acidic and alkaline media.^[^ ^134^ ^]^ Doping carbon‐based materials with heteroatoms such as S, O, P, B, N, etc. is an effective strategy to improve their ORR performance.^[^ ^60^, ^134^, ^135^ ^]^

**FIGURE 6:**
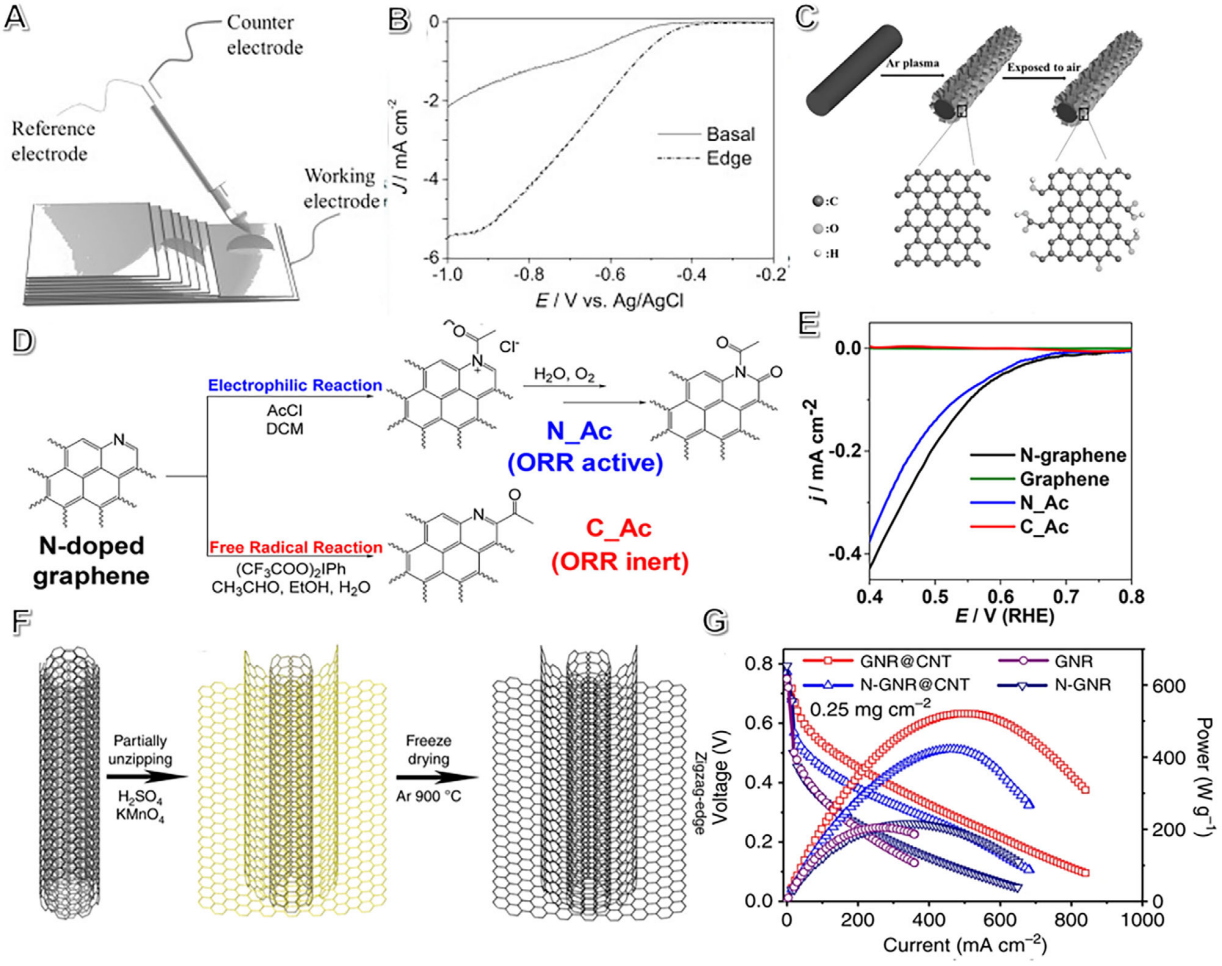
The role of metal‐free (carbon‐based) electrocatalysts on fuel cells. (A) Micro‐area electrochemical platform drawn in schematic image. (B) ORR performance edge and basal area for HOPG. Reproduced with permission.^[^ ^139^ ^]^ Copyright 2014, John Wiley and Sons. (C) Graphene diagram loading on carbon fiber etched with rich‐defect edge by plasma. Reproduced with permission.^[^ ^55^ ^]^ Copyright 2017, John Wiley and Sons. (D) Using an acetyl group, N‐doped graphene nanomaterial was prepared by the location‐specific modification of the pyridinic‐N and ortho‐C atoms. The modified pyridinic‐N and ortho‐C are labeled as N_Ac (blue) and C_Ac (red), respectively. (E) Polarization curves of ORR under the 0.1 M H_2_SO_4_ electrolyte in the saturated O_2_. (D, E) Adapted with permission.^[^ ^140^ ^]^ Copyright 2018, American Chemical Society. (F) Schematic diagrams of the GNR@CNT. (G) The relationship between the power density and cell voltage curves as a function versus current density for different catalysts. Reproduced with permission.^[^ ^141^ ^]^ Copyright 2018, Springer Nature Publishing.

Catalysts possess defects that can alter their electronic and chemical properties, thereby impacting their electrochemical performance, by changing the location and nature of defects.^[^ ^56^, ^136^ ^]^ This technique can enhance the activity and longevity of both non‐PGM and PGM‐based catalysts by adjusting the adsorption sites, and electronic structures.^[^ ^60^, ^137^, ^138^ ^]^ It is crucial to comprehend the connection between defective active sites and performance for designing robust electrocatalysts. Wang et al. found that heteroatoms play a crucial role in enhancing the ORR performance of adjacent carbon atoms.^[^ ^139^ ^]^ The self‐assembled micro‐area electrochemical platform demonstrated that the edge defects of graphene exhibit better ORR activity than the basal plane area (Figure 6A,B).^[^ ^139^ ^]^ Plasma etching has been found to be an effective method for producing graphene with high edge density and free from any dopants, which contributed to improved ORR activity by increasing adsorption energy for oxygen and decreasing the energy barrier.^[^ ^37^ ^]^ The process of in situ exfoliation has improved the number of graphitic edge defects on carbon fibers (Figure 6C), showing that defects and edges on the exfoliated graphene benefit the enhanced performance.^[^ ^55^ ^]^

Experimental and theoretical evidence is essential to identify the active sites of N‐doped carbon. As shown in Figure 6D,E, Zhou et al. developed a molecular probe approach to identify active sites by chemical dopants to a carbon‐based catalyst.^[^ ^140^ ^]^ An acetyl group was added to the ortho C atom or to the N position of the pyridine ring in N‐doped graphene using electrophilic and free radical reactions. At low pH levels, the carbon atom adjacent to pyridinic‐N serves as the active site. According to the comprehensive theoretical calculations, there are three main and undisputable conclusions. Firstly, the protonation of pyridine‐N is the crucial step without which it is extremely challenging to chemically adsorb O_2_ on the adjacent C. Secondly, the one and truly active site is the C atom of the C‐O bond, which is situated right next to pyridinic‐N. Finally, the enhanced ORR activity is undoubtedly attributed to the spin localization and positive charge at the active C atom, which leaves no doubt that N‐doped carbon species are the ORR active sites.^[^ ^140^ ^]^

Furthermore, researchers developed zigzag‐edged defect sites on graphene nanoribbons with carbon nanotubes as the backbone to improve performance in proton exchange membrane fuel cells.^[^ ^141^ ^]^ The GNR@CNT with a zigzag‐edged defective structure is fabricated by partially unzipping MWCNTs (Figure 6F), resulting in state‐of‐the‐art performance (520 mW mg^−1^) in various carbon‐based materials in PEMFCs (Figure 6G).^[^ ^141^ ^]^ They also synthesized N‐doped carbon nanomaterials with different N‐doped bonds and found that N_P_+N_G_ exhibited the best performance, which is comparable to commercial Pt/C ORR performance. DFT simulations verified that the structure of N_P_+N_G_ enhanced electron distribution, leading to a high charge density and high spin on the carbon matrix. These results present an exciting strategy for designing N‐doped carbon nanomaterials and confirm that the carbon atoms serve as the activity site for ORR.^[^ ^142^ ^]^

## 5 SYNERGISTIC STRATEGY OF METAL‐BASED CATALYST AND CARRIER MATERIALS

### 5.1 Synergistic strategies by surface functionalization: Anchor and physical encapsulation

Strengthening the interaction between catalysts and supports is a promising strategy for surface functionalization. Modifying organic ligands on the carbon supports via non‐covalent π‐π interactions is an effective approach.^[^ ^143^ ^]^ Surface ligands play a crucial role in downshifting the sintering of Pt‐based catalysts. Developing an organic‐inorganic hybrid structure is also an effective strategy to enhance the performance of Pt‐based catalysts.^[^ ^144^ ^]^ The non‐destructive functionalization method has a promising potential for application in PEMFCs.

Pt‐based catalysts are widely loaded on carbon‐based nanomaterials, which serve as the carrier in PEMFCs.^[^ ^8^, ^145^ ^]^ Carbon corrosion is a significant issue, particularly at the cathode, which can lead to the separation of Pt nanoparticles from the carbon matrix.^[^ ^146^ ^]^ To improve Pt‐based catalyst performance, researchers suggest strengthening the stability of the support, mitigating sintering, and inhibiting the dissolution of nanomaterials. Some researchers have found that the crystalline structure of carbon greatly resists oxidation rates. Graphene oxide, with its ordered structure, has even better corrosion resistance.^[^ ^147^ ^]^ Alternative strategies include using durable support materials, defect engineering on carbon surfaces, physically encapsulating Pt‐based catalysts with carbon, and strengthening interactions between carbon‐based carriers, proton conduction, and Pt‐based catalysts.

Using graphitized mesoporous carbon (GMPC) to support Pt catalysts resulted in a two‐fold durability increase compared to commercial Pt/C.^[^ ^148^ ^]^ Commercial carbon supports have insufficient interaction with Pt‐based catalysts due to the lack of long‐range order structure of the graphitic lattice.^[^ ^149^ ^]^ Stabilizing Pt‐based catalysts using decreased graphene oxide (rGO) as supports could extend their life. A composite material consisting of rGO and carbon black maintains the electrochemical active surface area (ECSA) of the ORR catalyst at 95% after 20,000 ADT cycles.^[^ ^150^ ^]^ Pt‐based catalysts are typically loaded on high surface area carbon black to avoid agglomeration, but their interaction is insufficient.

**FIGURE 7:**
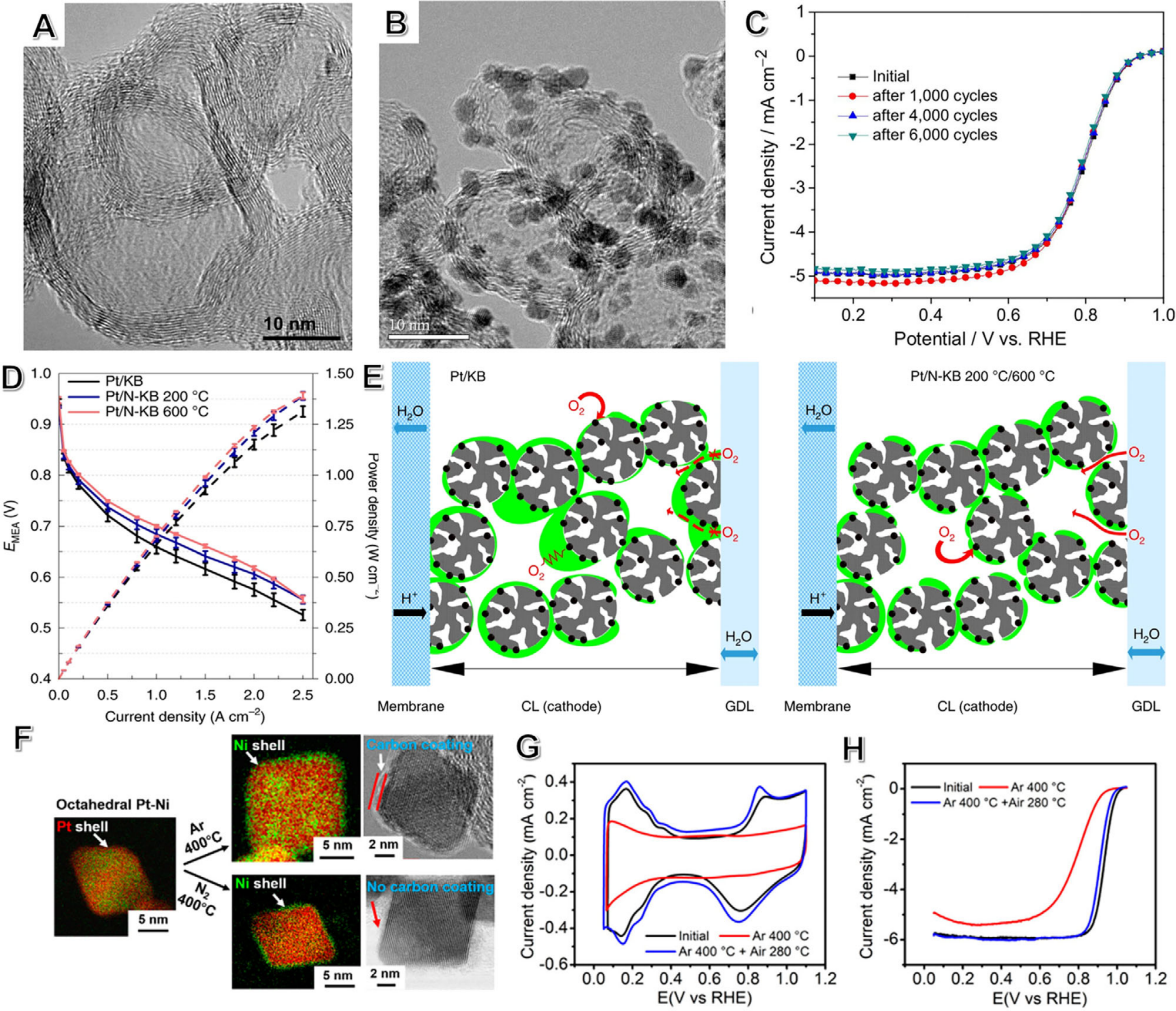
Synergistic strategies by surface functionalization: anchor and physical encapsulation. (A) TEM and (B) the HRTEM image of CNC and Pt/CNC; (C) Polarization curves of Pt/CNC before and after accelerated durability tests. (A–C) Reproduced with permission.^[^ ^151^ ^]^ Copyright 2014, Springer Nature. (D) Effect of N modification on ionomer distribution and performance in fuel cell. (E) N modification program of Pt/KB before and after thermal treatment. Reproduced with permission.^[^ ^152^ ^]^ Copyright 2020, Springer Nature. (F) HAADF‐STEMs and the corresponding HRTEM images in N_2_ and Ar under thermal treatment. (G) Cyclic voltammogram and (H) ORR polarization curve before and after different thermal treatments for the PtNi/C catalyst. (F–H) Reproduced with permission.^[^ ^153^ ^]^ Copyright 2019, American Chemical Society.

Hollow graphitic spheres (HGS) and carbon nanocages (CNC) are examples of 3D structures that have shown improved stability in fuel cells (Figure 7A–C).^[^ ^151^, ^154^ ^]^ The stability of CNC can be enhanced with a rich‐N content and high level of graphitization. The Pt/CNC (1000) catalyst showed almost no change in composition and morphology before and after the ADT test, demonstrating its high durability in PEMFC to ~3000 h. The significant stability is due to a high degree of graphitization of CNC, interaction with Pt NPs, and strong oxidation resistance. Similar mesoporous carbon spheres with 3D ordered arrays also inhibit Pt aggregation, enhancing durability.^[^ ^155^ ^]^

Pt‐based catalysts can be made more durable by modifying the carbon support. One way to do this is by creating defects on the carbon surface. Nitrogen‐doped or N‐functional carbon is often used to enhance performance by enhancing O_2_ dispersion and resistance to water agglomeration. Based on N‐modified carbon, the Strasser^[^ ^152^ ^]^ group recently demonstrated a catalyst synthesis strategy that exhibits excellent performance in PEMFCs (Figure 7D). Moreover, after carbon corrosion and N functionalization, the increase of mesoporous results in the opening of micropores and an increase in their specific surface area. The opened mesoporous structure allows for optimal mass transport, providing better oxygen accessibility to the Pt surface and aiding water management. To exhibit the superior performance of the Pt/N‐KB 600°C catalyst layer over the Pt/KB one under stoichiometric flow, polarization curves are presented in Figure 7D. Corrections for monopolar plate (MP) resistance or contact resistance of the MP/gas diffusion layer (GDL) RMP, MP/GDL demonstrated significant overall performance enhancement with the modified catalyst. The modified Pt/N‐KB 600°C catalyst layer offered advantageous transport properties that reduced voltage losses, especially in high current density regions. Consider Knudsen resistance, which increases for poor ionomer film distribution. The catalyst/platinum in fuel cells is not uniformly covered by the ionomer, which leads to partial blockage of the mesopore and the formation of a thick ionomer layer on the primary carbon particles. These regions represent additional resistance to oxygen transport through the catalytic layer. However, the presence of the N functional group changes the interaction between the polymer and the carbon carrier, promoting oxygen's mass transfer (Figure 7E). Additionally, nitrogen functionalization changes the hydrophobicity of carbon carriers, affecting water management.^[^ ^152^ ^]^ This has significant practical implications for commercializing PEM fuel cells, as mass transfer accessibility is greatly optimized due to reduced relative humidity requirements, reducing overall stack costs.

According to a report by Shao et al., pure carbon layer‐covered PGM‐based catalysts are not ideal for ORR.^[^ ^153^ ^]^ When the temperature goes beyond 350°C in Ar, it was found that an ultra‐thin carbon layer (∼1 nm) forms on the NP surface. STEM images confirmed the Ni‐rich PtNi/C after thermal treatment in Ar (Figure 7F). However, the ORR performance was obviously decreased than in air or in N_2_ (Figure 7G,H). This is because the coated carbon is not annealed in air or N_2_ environment. According to the results obtained from DFT calculations, the formed stable N‐C bond after thermal treatment may be the reason for the formation of a carbon layer in N_2_.^[^ ^151^ ^]^ Compared to the carbon‐encapsulated Pt catalyst, building a thin shell of N‐doped carbon seems like an effective way to enhance the performance of ORR. Kwon et al. synthesized platinum nanoparticles that were encapsulated in a thin layer of nitrogen‐doped carbon.^[^ ^156^ ^]^ This was achieved by thermally reducing a carbon nanofiber (CNF) that was coated with a Pt‐aniline complex. The annealing temperature was increased to improve the graphitization process, which resulted in a higher degree of stability. After 30 cycles, the loss in ECSA was within the DOE targets, with only a 15% excess. Moreover, the high corrosion resistance of the synthesized nanoparticles significantly contributed to the overall performance.

### 5.2 Strong interaction by surface functionalization: Chemical bonding

**FIGURE 8:**
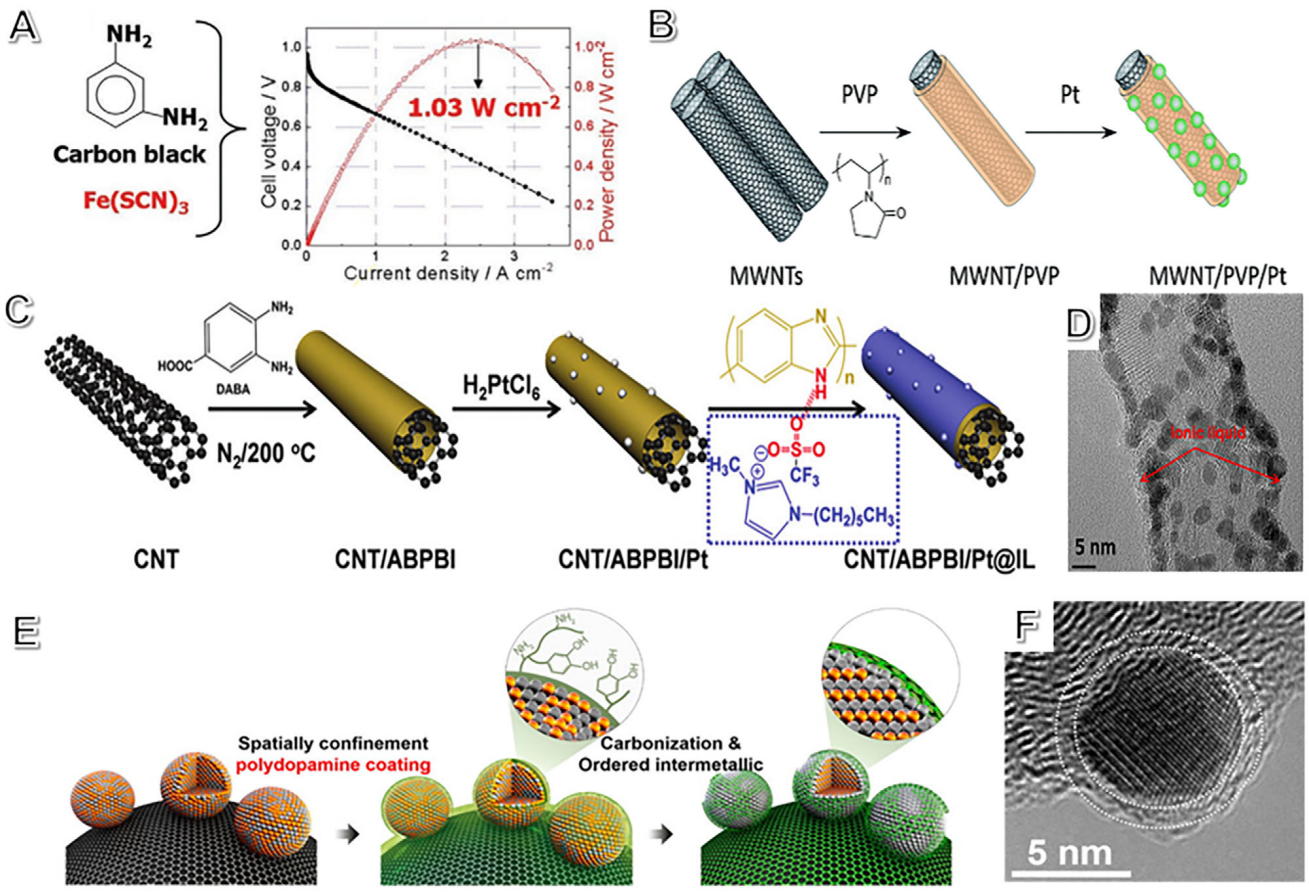
Strong interaction by surface functionalization: chemical bonding. (A) Fe/N/C ORR catalyst with doped sulfur synthesized using the poly‐m‐phenylenediamine, Fe(SCN)_3_ and carbon black (left); the relationship between the power density and polarization curve tested in a MEA with the S‐doped Fe/N/C cathode catalyst (right). Reproduced with permission.^[^ ^159^ ^]^ Copyright 2015, John Wiley and Sons. (B) Schematic illustration for the MWCNTs/PVP/Pt catalyst synthesis process with PVP; Reproduced with permission.^[^ ^164^ ^]^ Copyright 2013, Royal Society of Chemistry. (C) Schematic program of the as‐synthesized catalysts, (D) the corresponding CNT/ABPBI/Pt@IL image. Reproduced with permission.^[^ ^172^ ^]^ Copyright 2018, John Wiley and Sons. (E) Synthetic program, and (F) corresponding HRTEM image of ordered fct PtFe nanoparticle coated N‐doped‐carbon. (E, F) Reproduced with permission.^[^ ^173^ ^]^ Copyright 2015, American Chemical Society.

The carrier materials containing C/N and the polymer containing C/N can be used as high‐performance Pt‐based carrier materials by strengthening the strong interaction. The catalyst itself has a certain ORR active site and can further improve the overall performance through strong interaction such as anchoring, molecular bonding, physical packaging, etc.^[^ ^157^ ^]^ Lu et al.^[^ ^158^ ^]^ showed that halloysite‐templated N‐doped carbonaceous materials can obtain a 0.703 W cm^−2^ power density, surpassing the performance of the same material in a PEMFC. Wang's group revealed an S‐doped Fe‐N‐C catalyst that generated a power density of 0.94 W cm^−2^. Under 2 bar atmosphere, the power density first surpassed 1 W cm^−2^ (Figure 8A).^[^ ^159^ ^]^ In a bid to improve the performance of non‐precious metal catalysts at the cathode, researchers from the Shao group^[^ ^160^ ^]^ used a “ceria‐assisted” approach to increase the loading of Fe to 4.6 wt%, with a low catalyst loading of 1 mg cm^−2^, resulting in a PPD of 0.5 W cm^−2^.

For fuel cells, especially in HT‐PEMFC systems, commercial Pt/C catalysts used for ORR have significant issues, including poor performance, high Pt metal loading, and uneven dispersion. To address these problems, several modifications have been proposed, such as using Pt nanoparticles coated/anchored on graphitized carbon^[^ ^161^ ^]^ or MWCNTs,^[^ ^162^ ^]^ wrapping the polymers onto the catalyst support,^[^ ^163^, ^164^ ^]^ coupling the pyridine group with Pt, and coupling the pyridine group with Pt by using the MWCNTs as a carrier.^[^ ^165^, ^166^, ^167^, ^168^, ^169^ ^]^ These modifications can effectively improve the catalyst performance and should be considered to optimize ORR in HT‐PEMFCs. Yang et al. discovered that using a nanoporous carbon (Nano PC) support covered with pyridine‐containing PBI (PyPBI) significantly improves the CB/PyPBI/Pt performance.^[^ ^170^ ^]^ They prepared a catalyst NanoPC/PyPBI/Pt with 2.2 nm and tested it in practical HT‐PEMFC under 120°C. The results were impressive, with a power density of 342 mW cm^−2^, outperforming CB/PyPBI/Pt and commercial Pt/CB. The NanoPC/PyPBI/P catalyst also exhibited only a minimal loss of ECSA after 10,000 potential cycles, indicating improved stability and enhanced activity due to the unique nanoporous structure. The molecular bonding of PBI polymer offers robust synergistic strategies for ORR in HT‐PEMFCs.^[^ ^171^ ^]^ The Study confirms that 5 wt% Pt‐MoO*_x_*‐MWCNTs outperform Pt‐MWCNT when coupled with PBI as the membrane. The presence of Pt‐MoO*_x_* increases the concentration of O_ads_ species, improving the ORR performance. Encapsulating the polymer on the carrier enhances the performance of Pt‐based catalysts in HT‐PEMFCs by significantly enhancing proton conductivity at higher temperatures. Figure 8B shows that the use of poly(vinylpyrrolidone) (PVP) improves anchor sites, resulting in uniform Pt nanoparticle deposition on MWCNTs.^[^ ^164^ ^]^ This MWCNTs/PVP/Pt catalyst exhibits exceptional ORR durability, outperforming commercial Pt/C with a power density of 307 mW cm^−2^, which is 3.5 times higher. It also shows 15 times better durability, with ~40% ECSA loss after 150,000 cycles.

Effective proton conduction is critical to enhance HT‐PEMFC performance. Luo et al. found that doping carbon nanotubes (CNT) with ionic liquid (IL) in the Pt‐based catalyst (CNT/ABPBI/Pt@IL) improves the fuel cell's activity and stability without humidification at 120°C.^[^ ^172^ ^]^ This is achieved by enhancing the proton path (Figure 8C,D). The CNT/ABPBI/Pt demonstrated the smallest ECSA loss, highest current density, and remarkable stability, with a 512 mW cm^−2^ power density in the practice HT‐PEMFC at 120°C. The performance improvement is due to two reasons: (1) The distributed ionic liquid connects CNTs, promoting proton conduction and increasing three‐phase boundaries (TPBs) and decreasing the content of Pt. (2) Ionic liquids facilitate the transformation of electrons between N and Pt atoms, inhibiting Pt oxide formation.

Surface coatings prevent catalyst agglomeration, detachment, and dissolution. However, they reduce the number of active sites on the surface, limiting large‐scale transportation. Sun et al. have developed a technique to create fct‐PtFe nanoparticles coated with a shell with N‐doped carbon. This is done by thermally annealing surface‐coated polydopamine, improving ORR durability and activity.^[^ ^173^ ^]^ The N‐doped carbon shell, which is less than 1nm thick, protects the nanoparticles from sintering and reduces the Pt dissolution without affecting performance (Figure 8E,F). The ordered nanoparticles coated with N‐doped carbon show a significant improvement in durability and no noticeable morphology changes were observed after 100 h MEA testing.

### 5.3 Strong synergy between PGM catalysts and oxides/carbides carriers

Carbon materials are not durable enough for harsh long‐term tests in a real fuel cell, especially in HT‐PEMFC conditions.^[^ ^174^ ^]^ However, the metal oxide and carbon‐based metal derivative carrier materials like nanocrystalline tungsten carbide (WC),^[^ ^175^ ^]^ TiC,^[^ ^176^ ^]^ SiC,^[^ ^177^ ^]^ and TiC@TiO_2_,^[^ ^178^ ^]^ have shown high stability in the HT‐PEMFC. The TiC catalyst's overall catalytic activity remains unaffected by the presence of a surface oxide film, while the stability of ORR significantly improves. This has been confirmed by comparing the mass activities of ECSA and ORR of Pt_3_Pd/TiC@TiO_2_ and Pt_3_Pd/TiC catalysts.^[^ ^179^ ^]^ Furthermore, the carbon‐based metal derivative exhibits enhanced conductivity with temperature, making it an excellent alternative for catalyst support in HT‐PEMFC. Enhancing physical properties like the conductivity of metal oxides and carbon‐based metal derivative carrier materials in high‐temperature systems has become a new method to enhance electrocatalytic performance. By strategically altering the carbon support through techniques such as carbon doping and polymer encapsulation, the performance of PGM catalysts used for ORR can be drastically improved. This not only helps reduce the PGM catalyst loading on the cathode but also results in a significant reduction for the catalyst.

**FIGURE 9:**
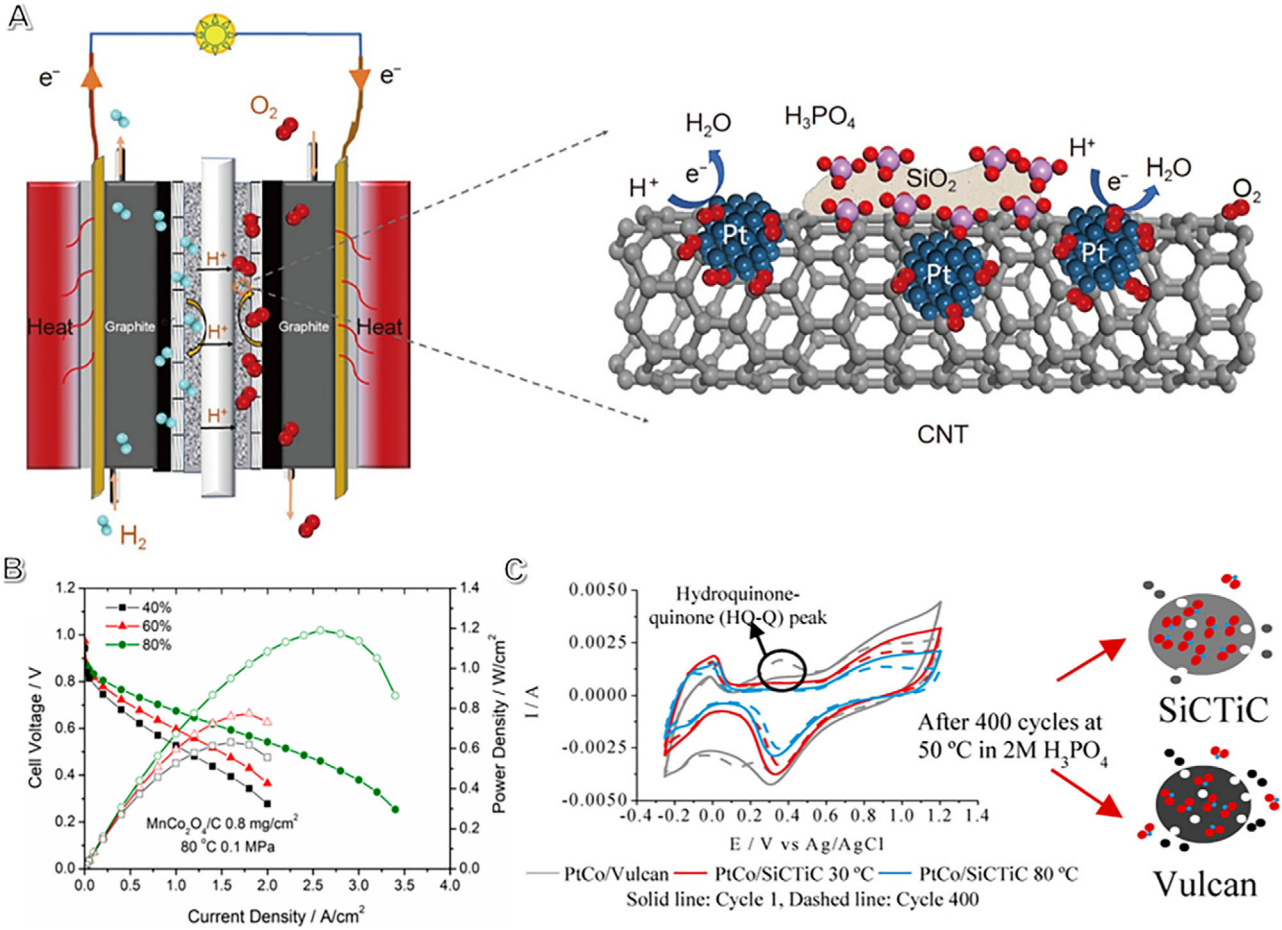
Strong synergy between PGM catalysts and oxides and metal carbide carriers. (A) Schematic diagram of silicon dioxide and carbon nanotubes working together as carrier materials for Pt nanoparticles in HT‐PEMFCs. Reproduced with permission.^[^ ^180^ ^]^ Copyright 2021, China Society of Chemistry. (B) MnCo_2_O_4_/C catalysts with varying metal loading are available for H_2_/O_2_ AMFC performance. Reproduced with permission.^[^ ^181^ ^]^ Copyright 2019, American Society of Chemistry. (C) The SiCTiC carrier exhibited excellent durability compared to the Vulcan support in HT‐PEMFCs. Reproduced with permission.^[^ ^184^ ^]^ Copyright 2017, American Society of Chemistry.

CNT@SiO_2_‐Pt catalysts have been developed for HT‐PEMFCs with platinum nanoparticles anchored on carbon nanotubes modified with silicon dioxide (Figure 9A)^[^ ^180^ ^]^. The SiO_2_ on CNT@SiO_2_‐Pt stimulates the adsorption of phosphoric acid, preventing platinum poisoning. SiO_2_ also creates a high way of proton conduction for the ORR process. CNT@SiO_2_‐Pt cathode has demonstrated exceptional stability, achieving a superior power density of 765 mW cm^−2^ (160°C) and 1,061 mW cm^−2^ (220°C).^[^ ^180^ ^]^ The temperature changes significantly improved the performance but did not change the composition and structure of the CNT@SiO_2_‐Pt catalysts. The transfer of charge between the support and the catalytic active site plays a crucial role in regulating the intensity of phosphoric acid adsorption on the active site. Based on this, the support can also facilitate the transfer of the adsorbed phosphoric acid from the catalyst to the support itself. This helps prevent the poisoning of the catalyst's active site.^[^ ^180^ ^]^ This breakthrough provides a new solution to the phosphate poisoning problem, by using oxides as carriers in HT‐PEMFCs.

Metal oxide‐based electrocatalysts have been applied in the research on AMFCs. However, compared to M‐N‐C catalysts tested in cell systems, metal oxide catalysts typically don't have high half‐wave potentials. Nonetheless, they perform exceptionally well when used in AMFCs. One study used carbon‐supported CoFe NPs to develop a multistage 3D electrode via the interaction between solid ionomer and carbon powder. Another study found that the fuel cell achieved the highest performance of 1.66 W cm^−2^ (IR‐corrected) among metal oxide electrocatalysts. MnCo_2_O_4_/C showed good performance in AMFCs, depending on the metal oxide loading on carbon. Increasing the metal oxide loading on carbon from 40 to 80 wt% resulted in a half‐wave potential of 0.84 V_RHE_ but showed a distinguished activity improvement. Due to more accessible active sites, the PPD demonstrated ~2.5 times enhancement due to the enhanced metal oxide loading (Figure 9B).^[^ ^181^ ^]^ Other catalysts, such as N‐C‐CoO*_x_* ^[^ ^182^ ^]^ and CoFe,^[^ ^183^ ^]^ have also been found to achieve PPDs above 0.6 W cm^−2^ in testing with H_2_/air. This shows non‐precious metal electrocatalysts can perform as well as Pt/C in AMFCs.

Researchers evaluated catalyst stability with 400 cyclic voltammetry cycles and found that the SiCTiC catalyst was more durable than Vulcan support (Figure 9C). The prepared catalysts performed better than PtCo/C and the PtCo/SiCTiC catalyst treatment under 80°C had the best result caused by the shorter Pt–Pt distance and higher alloy degree. The durability test in HT‐PEMFCs showed remarkable performance in both activity and stability.^[^ ^184^ ^]^

## 6 IN SITU CHARACTERIZATION SKILLS FOR THE ORR CATALYSTS

**FIGURE 10:**
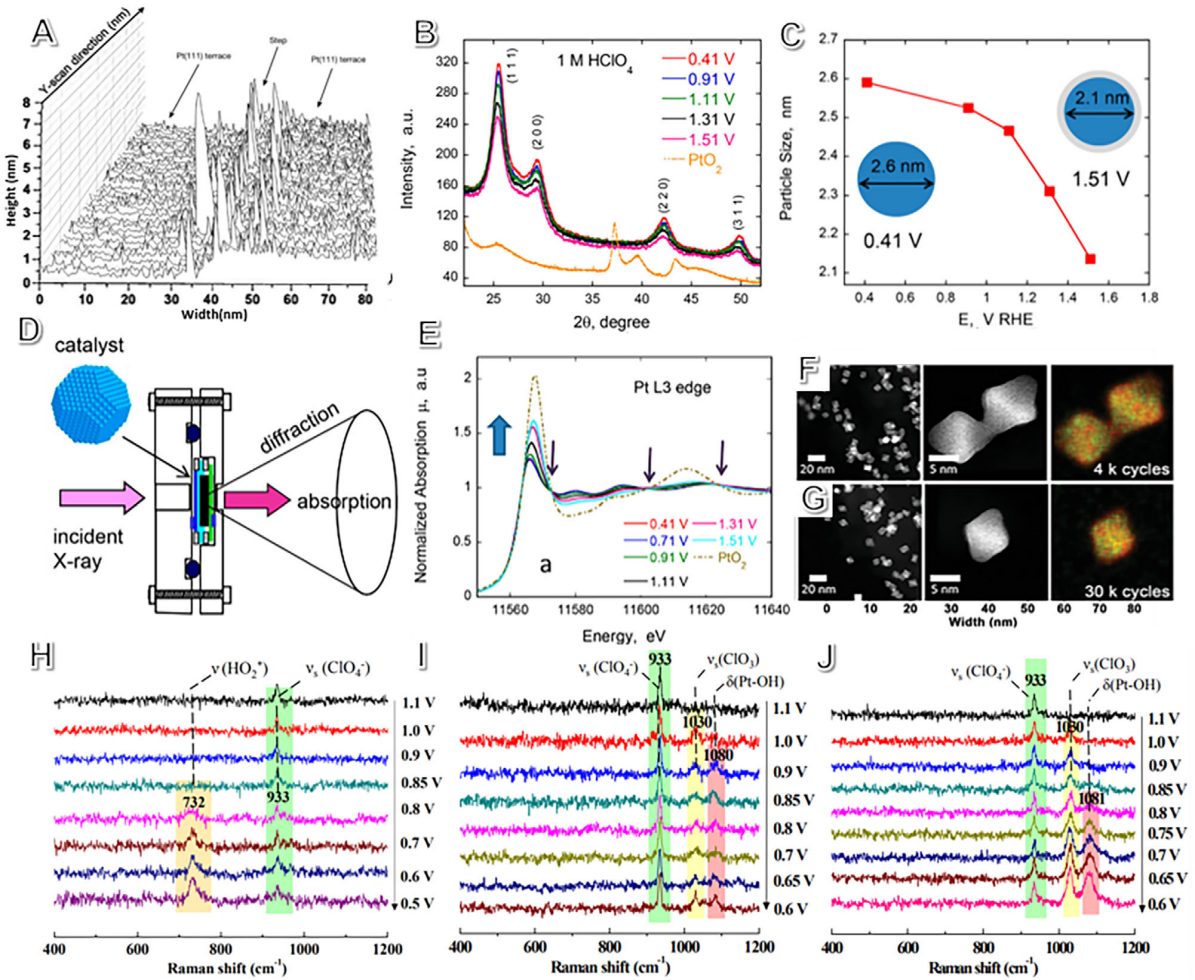
In situ characterization technique. (A) Line scan of STM over Pt(111) facet in PEMFCs cell. Reproduced with permission.^[^ ^191^ ^]^ Copyright 2017, Springer Nature. (B) In situ XRD test under various potentials for Pt/C catalyst, and (C) the corresponding function of nanoparticle size versus different potentials. (D) In situ XAS absorption cell and (E) the corresponding Pt L‐edge spectrum of PtCo under different potentials. (B‐E) Reproduced with permission.^[^ ^200^ ^]^ Copyright 2016, American Chemical Society. (F,G) HAADF‐STEM and EDX mapping images of Pt‐Rh‐Ni octahedral nanoparticles after different ADT tests. Reproduced with permission.^[^ ^89^ ^]^ Copyright 2016, American Chemical Society. In situ SHINERS spectra obtained from (H) Pt (111), (I) Pt (100), and (J) Pt (110) surfaces under the ORR environment. Adapted with permission.^[^ ^210^ ^]^ Copyright 2018, Springer Nature.

In situ techniques are crucial in obtaining accurate structure‐performance information on catalyst interaction in ORR electrochemical reactions. Electrocatalysts can be thoroughly investigated by employing various characterizations, such as aberration‐corrected transmission electron microscopy (ACTEM),^[^ ^142^ ^]^ X‐ray diffraction (XRD), Raman spectroscopy,^[^ ^37^ ^]^ and X‐ray photoelectron spectroscopy (XPS),^[^ ^185^ ^]^ electron paramagnetic resonance (EPR) spectroscopy,^[^ ^186^, ^187^ ^]^ and X‐ray absorption spectroscopy (XAS).^[^ ^188^ ^]^ The uncertain evolution process of catalysts in an actual fuel cell is still a complicated matter. Hence, to avoid similar issues in the future, it is crucial to develop in‐situ characterizations. When metal elements coordinate with the active intermediates, in situ STM can detect changes in electronic states. In situ XAS is also a favorable method to accomplish this task accurately. STM is a powerful tool for studying nanomaterials. It accurately detects surface topography and electronic state. Combining it with EC‐STM allows for time‐resolved detection of catalytic activity. This method has been used to map active sites on the surface of Pt (111), confirming that the terraces and steps both serve as active sites.^[^ ^189^, ^190^, ^191^, ^192^ ^]^ Additionally, EC‐STM can visualize the reconstruction of nanocrystals (Figure 10A).^[^ ^191^ ^]^ Bandarenka et al. developed a new way of identifying catalytic active sites on surfaces using EC‐STM. The method maps tunneling current noise to directly detect active centers,^[^ ^193^, ^194^ ^]^ making it ideal for ORR identification. Microscopic and macroscopic results validate its efficacy.^[^ ^195^, ^196^, ^197^, ^198^, ^199^ ^]^

XRD is a non‐destructive technique, widely used to determine materials' composition, structure, and physical properties. To obtain information about the evolution of the structure, the in situ XRD patterns could be used to test the aimed catalyst.^[^ ^200^ ^]^ Adzic et al. reported that, even when the potential increased from 0.41 to 1.51 V (vs NHE), the characterization peak of α‐PtO_2_ was not illustrated, as exhibited in Figures 10B and 10C. However, a drop in particle size was observed by using Sherrer's equation. Furthermore, the peak location of Pt (220) shifted negatively suggesting a longer distance of Pt‐Pt bonding arises for the α‐PtO_2_.^[^ ^200^ ^]^ As reported by Qian et al., In situ XRD can also be used to obtain information on the adsorption of oxygen intermediates during the ORR reaction process on the catalyst.^[^ ^201^ ^]^

Figure 10D displays the in situ XAS device diagram. This technique is helpful in determining the local structure of specific elements. The XAS spectrum is divided into three regions: the pre‐edge peak, X‐ray absorption near edge structure (XANES), and extended X‐ray absorption fine structures (EXAFS). The atomic structure of nanomaterials under different potentials can be identified by EXAFS,^[^ ^202^ ^]^ which reveals the local‐coordination environments such as bond length, coordination number, and degree of order (Figure 10E). Jiang's group performed in‐situ EXAFS analysis on Pt/C and PtCo/C catalysts within actual PEMFCs. Their findings revealed that after 30,000 ADT cycles, a PtO layer was covered on the Pt/C catalyst, causing a decrease in ORR performance due to active site blockage. In contrast, for the PtCo/C catalyst, a lengthening of the Pt‐Pt bond and a decrease in the coordination number of Pt‐Co suggested Co dissolution, resulting in decreased performance due to ligand effect and strain expansion.

Mukerjee et al. used in situ XAS to verify active sites and ORR pathways of Fe‐N‐C catalysts in alkaline and acidic mediums.^[^ ^203^ ^]^ The study found that the Fe‐N_4_ center site is active in the pathway 2e^−^ + 2e^−^ in alkaline conditions. In acidic conditions, a secondary site is needed; The carbon‐coated iron nanoparticles (Fe NP/C) neighboring to Fe‐N_4_ active sites, are required for the 4e^−^ process to further reduce the H_2_O_2_ intermediate. Liang et al. showed that the acidic ORR activity is lower because the doping of nitrogen atoms leads to protonation, reducing the extent of charge delocalization.^[^ ^204^ ^]^

HAADF‐STEM is a powerful tool with sub‐angstrom resolution, making it ideal for tracking atomic distribution and structural evolution.^[^ ^205^ ^]^ Used with in situ EDX, it's the go‐to for studying octahedral Pt‐Rh‐Ni NPs within actual fuel cells.^[^ ^89^ ^]^ Findings show NPs transformed into an alloy structure with a Pt‐rich shell after 30,000 potential cycles. As shown in Figure 10F,G, Rh‐inhibited Pt dissolution during the ORR process, enhancing oxidation resistance and vacancy formation energy.^[^ ^89^ ^]^

During the ORR process, the initial transfer of an electron to the adsorbed O_2_ molecule is considered the rate‐determining step.^[^ ^206^, ^207^ ^]^ Spectroscopic evidence has confirmed that the presence of an adsorbed superoxide anion (O_2_^−^) intermediate is formed on surfaces made of Pt and Au during ORR.^[^ ^208^, ^209^ ^]^ In acidic media, the Pt(111) surface has been shown to directly detect the presence of adsorbed HO_2_ intermediate, while in alkaline media, the ORR mainly benefits the formation of adsorbed O_2_^−^ intermediate on Pt (hkl) surfaces. Li et al. conducted a study employing a unique in‐situ shell‐isolated nanoparticle‐enhanced Raman spectroscopy (SHINERS) combined with DFT calculations and isotopic substitution experiments. It found that the Pt (111) surface directly detected adsorbed HO_2_ intermediate in acid media. However, only OH_ad_ was monitored on Pt (100) and Pt (110) surfaces (Figure 10H–J).^[^ ^210^ ^]^ These findings suggest that the ORR process occurs through an associative mechanism, where the proton and electron are simultaneously transferred to O_2_.

Using in situ characterization tools in practical PEMFC conditions can monitor the reaction mechanism and atomic structure evolution,^[^ ^205^, ^211^ ^]^ thereby aiding the research of structure‐performance correlation of catalysts and the development of advanced ORR catalysts.

## 7 SUMMARY AND PERSPECTIVE

To sum up, for PEMFCs, the engineering synergetic role of the crystal facet, defect site (coordination number/adsorption site effect), lattice strain, and vital synergetic role between the carrier and PGM catalysts were reported to improve the activity. Among the electrocatalyst engineering strategies, Co‐doping strategies for carbon‐based metal derivate and PGM catalysts could build a strong synergetic role at the interface and surface. Carbon catalysts, derivatives, and proton conductor polymers have gained attention in high‐temperature systems. Defective oxides and carbon‐based metal derivatives have shown an essential role in the application of fuel cells. In situ techniques are vital in obtaining the catalyst interaction in ORR. For instance, the 3D structural catalyst layer in MEAs can be detected, and the structure‐activity relationship can be tracked without the influence of pollutants, especially in actual PEMFCs.

Carbon‐based catalysts have massive potential for developing the cathodic carrier catalyst in fuel cells. By using defect engineering to alter the electronic structures and interface/surface properties, to precisely modify the adsorption ability of intermediates via redistributing charges. This subsequently promotes the electrochemical reaction rate. The use of doping engineering is a promising approach to increase positive charges and spin density, leading to enhanced activity. Carbon‐based metal‐free catalysts have a natural advantage of durability since there is no metal‐related corrosion and Fenton degradation. However, most carbon‐based catalysts illustrate lower performance compared to PGM catalysts. This results in a thicker MEA layer and a higher catalyst loading relative to PGM ones. Therefore, it is necessary to simultaneously develop both carbon‐based and PGM catalysts. Based on the discussion, we need to draw some important conclusions and vital perspectives as follows:

1. Catalyst design is crucial for PEMFCs. Resolving the problem of phosphoric acid poisoning is important in HT‐PEMFCs. The development of PGM alloy catalysts is key to improving the performance. For HT‐PEMFCs with a strong phosphoric acid environment, anti‐poisoning and corrosion‐resistant catalysts are imperative. Carbon‐based derivatives and metal oxides have shown potential value in both support materials and catalysts, especially in HT‐PEMFCs.
2. For the commercial prospects, a breakthrough is needed for the technology level of mass production with highly reproducible catalysts. Compared with the LT‐PEMFC system, the large scale of durable catalysts is distinguished and important for HT‐PEMFC. HT‐PEMFCs have a practical market for converting biomass, industrial exhaust gas, and waste liquid through electrocatalytic coupling and high‐temperature reaction systems.
3. Developing synthetic strategies with carrier materials and PGM‐based catalysts. The development of metal oxides and carbon‐based derivatives (for example, WC, TiC, SiC, etc.) illustrates the huge potential value in HT‐PEMFCs. For example: the synthetic strategy for constructing high‐index crystal facets with defects onto the surface to enhance performance. Enriching the base metal‐based inner core covering the high platinum alloy shell is crucial. Suitable lattice strain, favorable structural reorganization, and highly index active crystal facets are also critical. Finally, carbon‐based metal derivation catalysts with highly defective/active sites can greatly improve performance as carrier materials. Note that defect engineering is of great significance for developing high‐performance noble‐metal catalysts and non‐noble metal support materials, and the synergistic improvement of their performance.
4. Developing carbon‐based and PGM‐based electrocatalysts using in‐situ characterization skills and overcoming obstacles through theory and experiments.

Considering the synergetic role, both carbon‐based metal derivatives and PGM electrocatalysts are equally crucial for optimal ORR performance. Further development of effective catalysts is necessary to overcome PEMFC bottlenecks for commercial use.

## CONFLICT OF INTEREST STATEMENT

The authors declare no conflicts of interest.
